# A narrative review of the anatomy and function of the white matter tracts in language production and comprehension

**DOI:** 10.3389/fnhum.2023.1139292

**Published:** 2023-03-27

**Authors:** Ehsan Shekari, Nazbanou Nozari

**Affiliations:** ^1^Department of Neuroscience, Iran University of Medical Sciences, Tehran, Iran; ^2^Department of Psychology, Carnegie Mellon University, Pittsburgh, PA, United States; ^3^Center for the Neural Basis of Cognition (CNBC), Pittsburgh, PA, United States

**Keywords:** language, white matter, dorsal and ventral pathways, inferior longitudinal fasciculus, inferior fronto-occipital fasciculus, uncinate fasciculus, arcuate fasciculus, frontal aslant tract

## Abstract

Much is known about the role of cortical areas in language processing. The shift towards network approaches in recent years has highlighted the importance of uncovering the role of white matter in connecting these areas. However, despite a large body of research, many of these tracts’ functions are not well-understood. We present a comprehensive review of the empirical evidence on the role of eight major tracts that are hypothesized to be involved in language processing (inferior longitudinal fasciculus, inferior fronto-occipital fasciculus, uncinate fasciculus, extreme capsule, middle longitudinal fasciculus, superior longitudinal fasciculus, arcuate fasciculus, and frontal aslant tract). For each tract, we hypothesize its role based on the function of the cortical regions it connects. We then evaluate these hypotheses with data from three sources: studies in neurotypical individuals, neuropsychological data, and intraoperative stimulation studies. Finally, we summarize the conclusions supported by the data and highlight the areas needing further investigation.

## 1. Introduction

Detailed reviews exist of the role of cortical regions in language production and comprehension (e.g., Price, 2012; Kemmerer, 2019; Nozari, 2021). In recent years, however, interest has extended from uncovering the role of gray matter to how the interactions between different cortical regions give rise to language processing. A significant methodological development in this vein has been the study of white matter tracts, i.e., the pathways that connect various bodies of gray matter. The ultimate white matter map, the human connectome, represents a complex network of connections that forms the neurobiological basis of human cognition, including language processing. Compared to the study of gray matter, the study of white matter tracts in language processing is still in its infancy. New tracts are discovered, better anatomical descriptions of known tracts are offered, and new and more nuanced functions for each tract are frequently proposed in recent publications. The purpose of the current article is to present an up-to-date narrative review of the white matter tracts involved in language production and comprehension. We first present an overview of the computational architecture of comprehension and production, followed by a brief review of the role of the cortical regions in carrying out those computations. Next, we focus on each individual tract, its anatomical connections, and its hypothesized role(s) based on the cortical regions it connects. We then review the empirical evidence for and against such hypotheses, summarize the conclusions, and point out areas in need of further research.

## 2. The computational architecture of language production and comprehension

**Figure 1:**
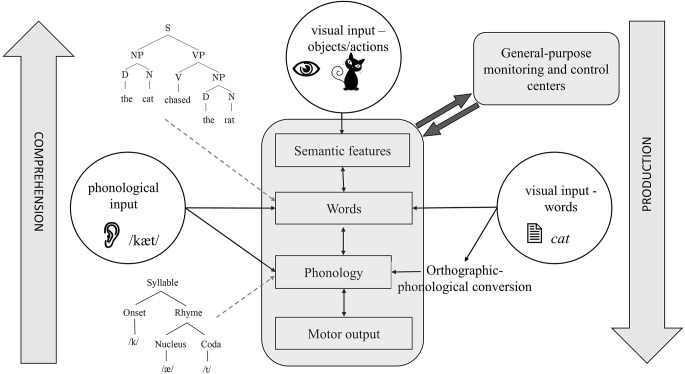
A schematic of the cognitive architecture of language production and comprehension. Depending on the input and direction of processing, different task architectures can be identified with this figure. For example, the central bubble, visual objects, shows the starting point of picture naming, which ends in motor output. The left bubble shows the starting point of auditory word repetition, which can be lexical or sublexical, depending on whether it activates words or phonemes, respectively. The right bubble shows the starting point of reading with visual orthographic input. Reading can also be carried out by directly activating words or through orthographic-phonological conversion. Some details are omitted to focus on showing how different task structures overlap in the language system.

Years of research and a large body of empirical evidence have been dedicated to uncovering the nature and levels of representations in language production and comprehension and the principles that govern these systems, leading to the proposal of sophisticated computational models (e.g., Dell, 1986; McClelland and Elman, 1986; Levelt et al., 1999). The gist is that the two systems have much in common (Figure 1). Production starts with formulating a message through the activation of semantic knowledge, and continues by activating lexical items, ordering them into a syntactic sequence, mapping each word onto its phonemes, activating the articulatory phonetic representations corresponding to the phonological plans, and ultimately executing speech motor commands. The system has a number of key properties: (1) spreading activation not only activates the target (e.g., cat) but also related representations (e.g., “dog”; Levelt et al., 1999; see Nozari and Pinet, 2020, for a review). (2) Activation is cascaded, meaning that activated non-target representations (e.g., “dog”) also activate their segments (e.g., /d/; Dell, 1986). (3) The system has some degree of feedback from later to earlier layers, i.e., phonemes /æ/ and /t/ in “cat” feedback to other words that share them (e.g., “mat”) and activate them (Dell, 1986; Rapp and Goldrick, 2000). These general properties are observed not only in spoken production but also in other production modalities such as handwriting and typing (e.g., Rapp and Fischer-Baum, 2014; Pinet and Nozari, 2018).

In many ways, comprehension can be viewed as an inverted version of production (see Figure 1). Here, the acoustic signal first activates the phonetic features. These features then activate phonemes, words, and ultimately semantic knowledge, translating sound into a meaningful message. While the nature of lower-level representations (articulatory-phonetic features vs. acoustic features) obviously differs between production and comprehension, most researchers agree that higher-level representations, e.g., words, semantic features, and syntactic structures are shared between the two (e.g., Warker et al., 2009; Nozari, 2020). Moreover, similar to production, comprehension also involves the co-activation of related non-target representations, cascading, and feedback (McClelland and Elman, 1986). These properties have several consequences for the studies of the neurobiology of language. Isolating various components (e.g., word representations) in cascaded systems is not easy. This is because activation can rapidly spread through the later layers of the system (e.g., Costa et al., 2009) while still converging on specific representations in earlier layers. The feedback from later to earlier layers further complicates the interpretation of events using a linear timeline. This, in turn, leads to difficulty in separating operations such as semantic-lexical activation and lexical selection (Riès et al., 2017). The good news is that despite the characteristics of cascading and interactivity, the evidence shows that, generally speaking, semantic-to-lexical mapping occurs earlier than lexical-to-phonological mapping (Dell, 1986; Rapp and Goldrick, 2000; Pinet and Nozari, 2023; see Dell et al., 2014, for a review). This so-called global modularity, despite local interactivity, has been a key factor in the success of neural studies in pinpointing individual operations to specific neural regions, but it is important to keep in mind that a clean demarcation between operations such as lexical activation and lexical selection and the neural regions responsible for the two is unlikely to be possible (Riès et al., 2017).

## 3. Neural underpinnings of language production and comprehension

Early studies of the neurobiology of language were mostly focused on defining the specific role of various cortical regions in language production and comprehension. This research has been largely successful in reconstructing the *language network*. The most widely accepted version of this network is Hickok and Poeppel’s (2007) dual-stream network. In this model, a largely bilaterally organized ventral stream is responsible for mapping sound to meaning. On the other hand, a predominantly left-lateralized dorsal stream maps the acoustic signal to articulatory motor commands. The two streams, thus, roughly carry out the operations related to comprehension and production, although equating the dorsal stream with production emphasizes production tasks that start with an available phonological sequence (e.g., auditory repetition). Production attempts that start from meaning (as most real-life conversations do) are likely to also involve a large portion of the ventral stream which carries out semantic-lexical processing. Relatedly, comprehension may entail production components, e.g., in the form of subvocal articulation (e.g., Price, 2010), making the contributions of the two streams to comprehension and production less modular. With that in mind, we briefly review the role proposed for different cortical regions in these two streams for language production and comprehension. In later sections, we will use this information to generate predictions about the role of white matter tracts connecting these cortical regions.

### 3.1. Semantic-lexical processing

Semantic processing is common to both comprehension and production, and as mentioned earlier, is unlikely to contain duplicate representations for these two systems. Detailed reviews of the semantic network exist elsewhere (e.g., Binder, 2007), but the gist is that there is an extensive network of distributed features (many of which are in the sensory-motor cortex) with potential “hubs” or convergent zones which represent unified concepts (Patterson and Lambon Ralph, 2016). There is disagreement about the degree to which such hubs contain lexical information vs. pure conceptual knowledge (e.g., Kemmerer, 2019), but computationally speaking, such hubs represent a graded translation of a massive, distributed network of semantic features into a much smaller space of phonological forms. Anterior and middle parts of the lateral temporal cortex (and less often the inferior temporal gyrus; ITG) have been the prime candidates for containing these semantic-lexical representations (Indefrey and Levelt, 2004; Binder et al., 2009; Walker et al., 2011). Of the two, the anterior temporal lobe (ATL) has been more strongly linked to semantic and the middle temporal gyrus (MTG) to lexical processing (Hickok and Poeppel, 2004; Indefrey and Levelt, 2004; Visser et al., 2010). In addition to the temporal cortex, parietal regions, especially the angular gyrus (AG), have been implicated in semantic processing (Binder et al., 2009; Price et al., 2015). However, unlike the temporal regions that largely represent individual objects and concepts, the parietal regions appear to be involved in integrative semantic processing, such as event representation (e.g., Binder and Desai, 2011; Ramanan et al., 2018). Finally, frontal regions are often activated during tasks that require semantic-lexical processing. This activation has been taken as representing a top-down boost, either for strengthening associations or for resolving conflict between competing alternatives (e.g., Thompson-Schill et al., 1997, 1998; Wagner et al., 2001). Both point to the concept of “semantic control” (as opposed to the simple activation of semantic knowledge) and mark the critical importance of the connections between temporo-parietal and frontal regions for semantic-lexical processing.

### 3.2. Processing of the acoustic signal

This function is primarily related to comprehension, although it also plays a role in regulating production through monitoring (e.g., Guenther, 2016). The regions usually implicated in the processing of the acoustic signal are the superior temporal gyrus (STG), Heschel’s gyrus, and the superior temporal sulcus (STS; e.g., Hickok, 2012). However, tasks that entail auditory discrimination such as changes to the phonetic category may also tap into other regions, such as the left dorsal pars opercularis in the IFG (e.g., Blumstein et al., 2005), indicating the importance of connections between the temporal auditory cortex and other regions.

### 3.3. Phonological processing

This function is hypothesized to be common to both production and comprehension, although not as uncontroversially as semantic-lexical processing. For example, while some researchers posit the existence of phonemes as distinct representations in perception (e.g., Hickok, 2012), others have questioned this assumption (e.g., Samuel, 2020). When assumed to be independent representations, the neural correlates of phonological processing have often been pinpointed to the posterior STG (pSTG), supramarginal gyrus (SMG), and sometimes posterior MTG (pMTG; e.g., Schwartz et al., 2012; Binder, 2015). It is noteworthy that phonological processing is often confounded with operations underlying phonological working memory (PWM), because keeping a phonological sequence active, say to output in production, relies on PWM. The latter is often localized to the inferior parietal cortex, especially SMG (e.g., Yue et al., 2018), and sometimes extends to the planum temporale (e.g., Buchsbaum and D’Esposito, 2008), although a frontal component has also been identified, which is hypothesized to mark the verbal rehearsal strategy related to keeping phonological forms active in working memory (e.g., Baldo and Dronkers, 2006).

### 3.4. Articulatory processing

This process is primarily related to speech production, although it is sometimes seen during comprehension as well (Price, 2010). The goal of this operation is to translate the phonological representations into motor commands. The neural regions proposed for this operation are the lateral and medial surfaces of the frontal cortex. GODIVA (see Guenther, 2016, for a history and complete review) is the most complete model of motor speech production and divides the process into a planning loop and a motor loop. The planning loop consists of the pre-supplementary motor area (preSMA) and left posterior inferior frontal sulcus (pIFS), and temporarily buffers the utterance before articulation. The motor loop consists of the supplementary motor area (SMA) and the ventral premotor cortex (vPMC) and executes the articulatory motor commands. A combined signal from the SMA and vPMC activates motor gestures in the ventral motor cortex (vMC), which drives the articulators (Guenther, 2016; Nozari, 2021).

### 3.5. Syntactic processing

The operations reviewed above are all involved in single-word processing, but speech often consists of phrases, sentences, and paragraphs. Although the body of literature probing the neural correlates of syntactic production is not small, pinpointing the neural substrates of syntax has been far from easy. For years, the inferior frontal gyrus (IFG), especially pars triangularis, was considered the main region involved in syntactic processing (Grodzinsky and Santi, 2008; Hagoort, 2014; Friederici, 2017; Matchin et al., 2017), primarily based on the neuropsychological evidence of patients with Broca’s aphasia suffering from agrammatism (Goodglass et al., 1968; Caramazza and Zurif, 1976; Goodglass, 1993). In line with this proposal, several high-powered lesion-symptom mapping studies linked IFG lesions to syntactic parsing deficits in comprehension (Wilson et al., 2010, 2011; Magnusdottir et al., 2013; Mesulam et al., 2015; Fridriksson et al., 2018). These were complemented with neuroimaging studies linking syntactic comprehension to IFG (Friederici, 2011, 2017; Hagoort, 2014). At the same time, more and more studies pointed out an even more prominent link between syntactic processing deficits and regions in the posterior temporal cortex (Dronkers et al., 2004; Wilson and Saygın, 2004; Baldo and Dronkers, 2007; Peelle et al., 2008; Pillay et al., 2017; Rogalsky et al., 2018; Wilson et al., 2018). In reviewing the neuroimaging data linking IFG to syntactic comprehension, Matchin et al. (2017) point out that the activation of IFG is almost always observed along with that of the posterior temporal lobe. The distinction is further complicated by the proposed involvement of the IFG in working memory and executive control processes that syntactic processing, in most cases, taps into (e.g., Rogalsky and Hickok, 2011; Nozari and Thompson-Schill, 2013, 2016; Nozari et al., 2014a, b; Arnold and Nozari, 2017). For this reason, some have proposed the posterior temporal cortex as a more critical region in syntactic processing than the IFG (Bornkessel-Schlesewsky et al., 2015; Pillay et al., 2017). A more nuanced proposal has been recently put forth by Matchin et al. (2017). The proposal emphasizes the different computational demands of syntactic processing in comprehension and production, which give rise to differential predictions regarding the role of certain regions in syntactic processing depending on the task. Specifically, Matchin et al. (2017) propose that in comprehension, auditory sequences in pSTG are decoded into hierarchical structures in pMTG, and are further connected to two semantic hubs, the ATL and the AG, representing the knowledge of objects and events, respectively (Binder and Desai, 2011). In production, the unstructured semantic information is turned into hierarchical propositions by the pMTG and passed on to the IFG’s pars triangularis for conversion into morphological chunks.

## 4. White matter tracts involved in language processing

As implied by their names, ventral and dorsal “streams” are more than just a collection of disconnected cortical regions. Rather, they mark connected pathways involved in semantic-lexical and phonological-motor processing, respectively. This rough division is a useful guide for identifying the white matter tracts potentially involved in language processing, although researchers sometimes differ in their assignment of tracts to streams, especially for multi-branch tracts that may encompass both streams. Generally speaking, the STG and Sylvian fissure mark the horizontal boundary between the ventral and dorsal streams (Hickok and Poeppel, 2007; Weiller et al., 2021). The ventral stream is often thought to include the inferior longitudinal fasciculus (ILF), the inferior fronto-occipital fasciculus (IFOF), the uncinate fasciculus (UF), the extreme capsule (EmC), and a branch of the middle longitudinal fasciculus (MdLF; Saur et al., 2008; Wong et al., 2011; Dick and Tremblay, 2012; Yang et al., 2017; Weiller et al., 2021). The dorsal stream contains the bulk of SLF, consisting of SLF-I, SLF-II, SLF-III, and SLF-tp, a part of the SLF that connects temporal and parietal lobes. Some researchers also consider the arcuate fasciculus (AF) to be another branch of the SLF. Finally, a more recently discovered tract, the frontal aslant tract (FAT; Catani et al., 2012, 2013), lies in the anteriormost part of the dorsal tract. In addition to these, there are a few other small tracts that are not frequently included in studies of white matter for language, such as the operculo-premotor fascicle (OpPMF) connecting the pars opercularis to the premotor region and trianguloorbitaris system (TrOrS) connecting the pars triangularis to the pars orbitalis. These tracts are usually difficult to identify in fiber-tracking studies because of their small size and their overlap with SLF III (Lemaire et al., 2013). Although some have suggested a role of these tracts in language processing based on their anatomical connections (Lemaire et al., 2013; Mandonnet et al., 2016), functional data on these tracts are currently sparse. Therefore, we do not include them in this article.

In what follows, we discuss the above-mentioned tracts individually (or sometimes in pairs for comparison). We first review the anatomy of the tract and the cortical regions it connects, based on which predictions about its function can be generated. We then review the empirical evidence regarding the role of the tract, with a heavier focus on its involvement in language processing, and discuss the extent to which the current evidence supports the predictions.

### 4.1. Methodological preview

Empirical evidence for studying white matter connectivity comes from several different sources. A precise method for studying the anatomy of white matter, used by early anatomists, is post-mortem fiber dissection. This technique entails the peeling of the white matter tracts from the brain and displaying their 3-dimensional structure. The complex and cumbersome procedures required for the preparation of the brain tissue for fiber dissection, together with the emergence of non-invasive methods, have decreased the popularity of this approach, although its precision for studying the subcomponents of white matter tracts has led to renewed interest in its revival in recent years (Martino et al., 2013; Kalyvas et al., 2020). Another precise method is autoradiography, an imaging technique using radioactive tracers, which allows for clear tracing of the origins and termination points of neural pathways (Cowan et al., 1972). Due to toxicity, *in vivo* autoradiography is not an option in humans but has been a widely used technique for the identification of white matter pathways in primates, such as Rhesus monkeys (Schmahmann and Pandya, 2006; Schmahmann et al., 2007).

Although precise, neither of the two methods described above is practical for studying white matter structures in living humans. A much more popular and widely used non-invasive technique for analyzing white matter in humans is diffusion MRI (dMRI). The most frequent method for analyzing the data is diffusion tensor imaging (DTI; Basser et al., 1994; Mori and Zhang, 2006; Mukherjee et al., 2008; Craddock et al., 2013). This technique relies on the displacement of water molecules in the tissue (Basser et al., 1994). The anisotropic nature of water molecules forms the basis of the quantitative DTI measures: while water molecules diffuse more freely along the axons, myelin sheaths restrict the diffusion of molecules perpendicular to the axonal lines. This difference in the diffusion rate can be used to reconstruct the white matter architecture (Basser et al., 1994; Le Bihan et al., 2001; Alexander et al., 2007). DTI is informative in uncovering the white matter structure in both neurotypical individuals and clinical populations. It uses a number of metrics, the most common of which are fractional anisotropy (FA), mean diffusivity (MD), radial diffusivity (RD), and axial diffusivity (AD). FA varies between 0 and 1 and measures the degree of diffusion anisotropy. When diffusion is unrestricted (or equally restricted in all directions), FA is 0. When, on the other hand, diffusion is fully restricted along one axis, FA is 1. Therefore, in gray matter in which the motion of molecules is in all directions, FA is low. In the white matter, the perpendicular motion of molecules is restricted by the myelin sheath and therefore diffusion is anisotropic and FA approaches 1 (Le Bihan et al., 2001; Alexander et al., 2007). Mean diffusivity (MD) measures the overall diffusivity in the tissue. Similar to FA, it is sensitive to the barriers of diffusivity but, unlike FA, it is insensitive to the direction of the diffusion, i.e., it measures the rotationally invariant magnitude of water diffusion in the tissue (Le Bihan et al., 2001; Alexander et al., 2007). In case, of lesions to the white matter, MD increases (Madden et al., 2012). AD and RD indicate the direction of diffusivity. AD describes the magnitude of diffusion parallel to axons, and is a specific marker of axonal degeneration, whereas RD describes the diffusivity perpendicular to axonal fibers and is more sensitive to demyelination (Le Bihan et al., 2001; Alexander et al., 2007). Thus, different measures may be useful in detecting different deficits. For example, relatively pure myelin deficits that are undetectable with FA, often lead to a modest increase in RD (De Erausquin and Alba-Ferrara, 2013). It is important to note that DTI is a correlational technique, meaning that although an association can be established between certain structural properties of the white matter tracts and behavior, it may not translate into a causal role of the tract in generating a certain behavior. Nevertheless, gaining insight into the structural connectivity by DTI, combined with resting-state functional connectivity by fMRI could improve our understanding of network structures underlying brain functions (Skudlarski et al., 2008; Greicius et al., 2009).

Another popular technique for studying white matter is voxel-based lesion-symptom mapping (VLSM) or its related technique voxel-based morphometry (VBM; Bates et al., 2003; Mechelli et al., 2005). These techniques are heavily employed in neuropsychological work and define a statistical relationship between lesion (measured in voxels) and behavioral impairment (Harvey and Schnur, 2015; Gleichgerrcht et al., 2017; Faulkner and Wilshire, 2020). The rationale is that if damage to a tract leads to an impairment in behavior, the tract is likely to play a critical role in carrying out that behavior. Although the results are less prone to interpretation as an epiphenomenon compared to associative measures, it is still difficult to pinpoint a function to a single tract. The reason is that impaired behavior as a function of a lesioned tract may reflect the disruption of a network, another part of which may play an even more critical role in the behavior than the lesioned tract itself.

Unlike DTI and VLSM/VBM, direct stimulation of white matter (DES; Mandonnet et al., 2010; Duffau et al., 2014; Duffau, 2015) is an invasive methodology that allows for real-time direct functional mapping of white matter tracts intraoperatively. It involves applying localized electrical stimulation to cortical and/or subcortical areas *via* either a monopolar or bipolar electrode to produce a transient inhibition or excitation in function. The technique may be employed in both an asleep and awake patient. In an asleep patient, where eliciting an overt behavioral response is not possible, electrophysiological measures such as electromyograms or visually evoked potentials are sometimes obtained. In awake patients, changes to performance on a behavioral task is often measured as a function of stimulation. Since the technique involves direct manipulation of the tissue, a change in behavior can be more readily translated into a causal role for the tissue in generating that behavior. Finally, we include some developmental studies in our review. It is important to note that the network for a cognitive function may change over the course of development, but including developmental data in the review allows us to examine whether a certain tract has been implicated in* learning* a function, even if after learning the function may continue to operate without strong dependence on that tract.

The techniques named above have identified three main types of white matter pathways: *projection*, *commissural*, and *association fibers* (Gottlieb and Cowan, 1973; Schmahmann et al., 2007; Wedeen et al., 2008; Zhang et al., 2010). Projection fibers are ascending and descending fibers which connect the cortex with the brainstem, cerebellum, and spinal cord. The most well-known projection fiber in the brain is the internal capsule. The commissural fibers are axons that connect the two hemispheres (Catani et al., 2002). The main commissural fibers are the corpus callosum, the anterior commissure, and the posterior commissure. Association fibers are axons which connect cortical areas within the same hemisphere. Long and short association fibers connect distant and adjacent areas, respectively (Guevara et al., 2020). Here, we focus on the role of association fibers in language processing.

## 5. ILF and IFOF

### 5.1. Anatomy

**Figure 2:**
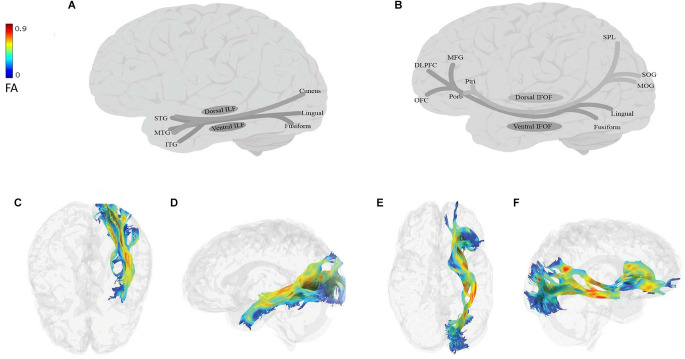
Anatomy of ILF (**A**: schematic; **C**: tractography, axial view; **D**: tractography, sagittal view) and IFOF (**B**: schematic; **E**: tractography, axial view; **F**: tractography, sagittal view). All tractography images have been extracted in the following way: DTI data of a 45-year-old neurotypical male were used for fiber-tracking of the language tracts. ROIs were defined automatically based on the anatomical atlas loaded into the DSI studio program. The angular threshold was 90 degrees. The step size was 0.98 mm and 10,000 tracks were placed. Fiber trajectories were smoothed by averaging the propagation direction of the 30% of previous directions, and tracts shorter than 20 mm were discarded. DLPFC, dorsolateral prefrontal cortex; FA, fractional anisotropy; ITG, inferior temporal gyrus; MFG, middle frontal gyrus; MTG, middle temporal gyrus; MOG, middle occipital gyrus; OFC, orbitofrontal cortex; Ptri, pars triangularis; Porb, pars orbitalis; SOG, superior occipital gyrus; SPL, superior parietal lobe; STG, superior temporal gyrus.

The ILF and IFOF are the two major fiber tracts connecting the occipital lobe to the anterior regions (temporal and frontal lobes; Figures 2A–F). After some initial controversies about the nature of these pathways, DTI, electrostimulation, and non-human primate studies have now established that ILF and IFOF are long association—and not projection—fibers (Mettler, 1935; Seltzer and Pandya, 1984; Catani et al., 2002, 2003; Wakana et al., 2004; Schmahmann and Pandya, 2006; Mandonnet et al., 2007; Hua et al., 2008; Oishi et al., 2011). There has also been much debate on whether ILF and IFOF are indeed two separate tracts or whether IFOF is the continuation of ILF into the MdLF, EmC, and UF (Schmahmann and Pandya, 2006). This debate is important, in part because of the different endpoints of these tracts: ILF ends in the temporal pole, whereas IFOF ends in the frontal cortex. Therefore, the existence of IFOF as an independent tract would point to direct connections between the occipital and frontal lobes. Unlike studies of non-human primates (Mettler, 1935; Schmahmann and Pandya, 2006; Yeterian et al., 2012), DTI studies in humans consistently support the separation of these two tracts (Catani et al., 2002, 2003; Wakana et al., 2007; Hua et al., 2008; Oishi et al., 2010; Holl et al., 2011; Thiebaut de Schotten et al., 2011; Turken and Dronkers, 2011; Caverzasi et al., 2014). Given our focus on the role of these tracts in human cognition, we will follow the DTI findings and assume that they are separate tracts, but discuss them together to compare and contrast when possible.

The ILF (Figures 2A,C,D) is a large multilayer fiber tract connecting the occipital cortex with the anterior temporal lobe (Catani et al., 2002, 2003; Panesar et al., 2018; Sali et al., 2018; Zemmoura et al., 2021). Its dorsal component originates from the cuneus and projects to the superior and middle temporal gyri. Its ventral component originates from the lingual and fusiform gyri, and projects to the superior, middle, and inferior temporal gyri (Latini et al., 2017; Panesar et al., 2018; Sali et al., 2018; Zemmoura et al., 2021). The IFOF (Figures 2B,E,F) is also a large multilayered tract originating from the occipital and occipitotemporal, and parietal regions. It runs through the extreme and external capsules and terminates in anterior frontal areas, including the IFG and the dorsolateral prefrontal cortex or DLPFC (Duffau, 2015; Conner et al., 2018a). The IFOF has two components: (i) a superficial dorsal component, which connects the pars triangularis and orbitalis with the superior parietal lobe and the posterior portion of the superior and middle occipital gyri; and (ii) a deep ventral component, which connects the posterior portion of the inferior occipital gyrus and the posterior basal temporal region with three different areas in the middle frontal gyrus (MFG), the DLPFC and the orbitofrontal cortex (Martino et al., 2010; Sarubbo et al., 2013; but see Thiebaut de Schotten et al., 2011; Wu et al., 2016).

### 5.2. Function

Given the occipital origin of both tracts and the links they provide to temporal, parietal, and frontal regions, it is hardly surprising that they have both been implicated in operations that depend on visual perception, such as object and face recognition and spatial attention. In keeping with these predictions, DTI studies have shown a role for ILF in object recognition (e.g., Mandonnet et al., 2009; Ortibus et al., 2012), scene recognition (e.g., Tavor et al., 2014), and face recognition (e.g., Tavor et al., 2014; Hodgetts et al., 2015). Complementing these, are reports of prosopagnosia after the disruption of right ILF (e.g., Thomas et al., 2009; Valdés-Sosa et al., 2011; Grossi et al., 2014), and the induction of visual hemiagnosia after the stimulation of the right ILF (Coello et al., 2013), further pointing to a critical role of the ILF in face and object recognition.

Evidence for the role of IFOF in face perception is mixed. Some have linked the integrity of IFOF to face perception in prosopagnosia and neurotypical adults (e.g., Thomas et al., 2008; Valdés-Sosa et al., 2011) but others have explicitly shown that such a relationship is limited to the right ILF (e.g., Scherf et al., 2014). In contrast, and perhaps due to its stronger connections with the parietal regions, IFOF is more heavily implicated in spatial attention. For example, a decreased FA in the right IFOF in stroke survivors is associated with visual neglect (Urbanski et al., 2011; Toba et al., 2018). Similarly, Herbet et al. (2017a) showed that subcortical stimulation of right IFOF in glioma patients can cause spatial neglect. Both ILF and IFOF have also been linked to the processing of facial emotions (Baggio et al., 2012; Multani et al., 2017).

### 5.3. Links to language

Aside from a general role in visually guided behavior, connections to a specific part of the temporal cortex, the visual word form area (VWFA), suggest a potential role of these tracts in a specific language task, i.e., reading and perhaps writing. ILF has been identified as one of the three major tracts associated with the VWFA, with SLF and the vertical occipital fasciculus (vOF) as the other two (Chen et al., 2019). vOF is a fiber bundle connecting dorsolateral and ventrolateral visual cortices but is rarely mentioned in studies of the matter tracts, most likely because of its overlap with the dorsal parts of ILF and IFOF (Yeatman et al., 2014). It is thus important to keep in mind that some of the functions attributed to ILF and IFOF in reading may actually be carried out by vOF. For example, studies that dissociate the vOF from the ILF and IFOF showed that this tract is related to literacy development in children (Broce et al., 2019; Grotheer et al., 2021).

Various studies have linked reading or reading deficits to ILF, IFOF, or both (Horowitz-Kraus et al., 2014; Sarubbo et al., 2015; Vandermosten et al., 2015; Zhao et al., 2016; Arrington et al., 2017; Broce et al., 2019; Kumar and Padakannaya, 2019; Grotheer et al., 2021). For example, after controlling for several factors, including age, gender, IQ, the overall development of the white matter, and phonological skills, Broce et al. (2019) showed that the properties of the left ILF and right IFOF were predictive of early literacy skills in 5–8-year-old children. The left ILF was, however, strongly associated with phonological awareness. In a study comparing typically developing children with children with dyslexia, Zhao et al. (2016) showed that IFOF was significantly less left-lateralized in the dyslexic group, and the degree of lateralization was correlated with reading abilities (see also Steinbrink et al., 2008; Carter et al., 2009; Yeatman et al., 2012; Vandermosten et al., 2015; Su et al., 2018a; Vanderauwera et al., 2018; see Vandermosten et al., 2012 for a review).

The Left ILF’s critical role in orthographic processing was demonstrated in a study of 67 individuals with brain damage, in whom a PCA-derived orthographic index (after regressing out non-orthographic tasks) correlated significantly with left ILF’s integrity, even after controlling for other confounding factors (Wang et al., 2020; see also Su et al., 2018b; Farah et al., 2020). Reports from awake craniotomy studies also confirm the link between these tracts and reading/writing abilities. In a study of glioma patients undergoing awake surgery, Sarubbo et al. (2015) found a close correspondence between the spatial distribution of alexia and that of the ILF (see also Epelbaum et al., 2008; Gil-Robles et al., 2013; Enatsu et al., 2017 for demonstrations of ILF stimulation disturbing reading). Similarly, in a patient undergoing surgery for left inferior parietal glioma, Motomura et al. (2014) applied subcortical stimulation to IFOF and induced transient alexia and agraphia, suggesting a critical role of IFOF in these operations.

The literature above clearly links the ILF and IFOF to language processing through reading. There is, however, evidence that these tracts contribute to language processing in more fundamental ways, namely through their involvement in semantic-lexical processing. General evidence for this claim comes from studies linking comprehension abilities to the ILF, IFOF, or both (e.g., Del Tufo et al., 2019), or demonstrating their abnormalities in impaired semantic processing in neuropsychological disorders (e.g., Whitwell et al., 2010; Botha et al., 2015; D’Anna et al., 2016; Ivanova et al., 2016; Surbeck et al., 2020). There have also been attempts at pinpointing the function(s) of these tracts more precisely. Anatomically, there are two reasons to expect a contribution of these tracts to lexical semantic processing: (a) traversing through the length of the temporal lobe, they connect areas that are clearly implicated in storing conceptual, lexical, and auditory representations, and might thus point to a role in semantic and lexical *retrieval*. (b) Connections between the temporal lobe (especially MTG) and frontal (and some parietal) regions point to a potential role in semantic and lexical *control.* The main difference between the two is that the former includes any situations that require activation of semantic-lexical concepts, while the latter selectively involves situations that include either high competition or weak association (Martin-Chang and Levy, 2006; Nozari et al., 2016a). We will return to this point at the end of this section.

The standard test of pure semantic processing is usually a test such as the Pyramids and Palm Trees Test which assesses semantic relations without the need to appeal to lexical labels. There are indeed reports of a correlation between scores on such tests and IFOF in particular (de Zubicaray et al., 2011; Moritz-Gasser et al., 2013; Mirman et al., 2015; Herbet et al., 2017b). But the task is heavily influenced by visual processing abilities that are also linked to ILF and IFOF. To ensure the pure contribution of these tracts to semantic processing independently of visual processing, it is important to use other modalities (e.g., the auditory modality). This often means that some of the tasks entail lexical items, which makes the extraction of pure semantics difficult. Fortunately, based on the earlier discussion of the stages of production, such a clear-cut distinction is perhaps neither necessary nor extremely useful in understanding the neurobiology of language: lexical-semantic representations are representations necessary for the mapping of distributed semantic features onto phonological representations, and likely include multiple layers of representations that gradually move from unifying semantic features to representing the more formal aspects of concepts. We will thus focus on studies providing converging evidence from multiple tasks on the involvement of the ILF/IFOF in lexical-semantic processing.

Faulkner and Wilshire (2020) used VLSM in 63 postoperative tumor patients and found a correlation between lesions in the territory of left ILF and semantic-lexical mapping, which they computed as a function of semantic and omission errors in picture naming, as well as performance on a category probe task after regressing out phonological fluency scores (note that the use of probabilistic maps in that study makes disentangling from close tracts such as left IFOF very difficult). Using diffusion-weighted imaging, Jarret et al. (2022) studied picture naming in 37 neurotypical adults and reported IFOF as a direct pathway for lexicosemantic processing, while ILF and UF were reported as indirect pathways for the same function. In a stud of 55 right-handed older adults, de Zubicaray et al. (2011) used PCA to extract what they referred to as a “semantic memory” component, based on six cognitive tests (Pyramids and Palm Trees, Boston naming, PALPA sentence–picture matching, Category fluency, WAIS-III information, and WAIS-III similarities). They then demonstrated a correlation between this measure and FA values in left IFOF and UF. In a VLSM study of 43 individuals with chronic left-hemisphere stroke, Griffis et al. (2017) showed that lesions in the ILF and IFOF (as well as AF) were associated with poorer performance in picture naming. This may be interpreted as a problem in extracting the visual semantic information, but these lesions were also predictive of poor verbal fluency and auditory semantic decisions, two tasks that do not require visual processing, but instead, semantic-lexical processing. In another large-scale VLSM study of 99 individuals with chronic stroke, Mirman et al. (2015) used a large battery of cognitive tests and used factor analysis to extract three main factors: semantic recognition (encompassing both verbal and non-verbal comprehension), speech recognition (auditory processing of verbal materials), and speech production (phonological and articulatory phonetic encoding). Of the three, semantic recognition was associated with lesions in the IFOF (as well as UF and anterior thalamic radiation or ATR). In another large-scale study of 76 individuals with brain damage, Han et al. (2013) used visual and auditory variants of naming and semantic judgment tasks and found impaired performance to correlate with the FA value of IFOF, as well as the left ATR and UF, after controlling for lesion volume and refuting the influence of gray matter, non-semantic operations (e.g., oral repetition) and numerical cognition.

Together, these studies demonstrate that these tracts play a role in semantic-lexical processing, distinct from other aspects of language processing, such as processing phonological information. The involvement of ILF and IFOF in semantic-lexical processing naturally predicts a role for them in language production from meaning. In line with this prediction, Grossman et al. (2013) studied 15 individuals with nonfluent aphasia and reported a correspondence between the integrity of the fronto-occipital white matter (most likely the IFOF), as well as the UF, and the mean length and well-formedness of utterances. Several studies have more sharply focused on the relationship between these tracts and speech errors in picture naming. If these tracts play a critical role in mapping semantics to lexical items during word production, their disruption should lead to an increase in certain error types but not others. Specifically, we would expect an increase in lexical errors (including semantically related and unrelated words) and possibly omissions (due to the inability to retrieve the correct semantic concept or lexical item). The empirical data are aligned with this prediction: in a study of 32 participants with chronic post-stroke aphasia, McKinnon et al. (2018) found a close correspondence between the number of semantic paraphasias and axonal loss in the ILF. Similarly, in a combined navigated transcranial magnetic stimulation (nTMS) and DTI of 10 patients with brain tumors, Raffa et al. (2016) found a link between semantic errors in picture naming and the ILF and IFOF (see Sarubbo et al., 2013 for a similar link between semantic paraphasias and IFOF). Finally, Stark et al. (2019) studied speech errors of 120 individuals with chronic left-hemisphere stroke in a picture naming task, and also administered a test of connected speech using picture description. In both picture naming and connected speech, unrelated errors were linked to the ILF and IFOF, and in connected speech, semantic paraphasias were also linked to these two tracts (it is noteworthy that SLF was implicated in all of these cases as well).

Complementing these, are studies of intraoperative stimulation of the ILF/IFOF. The majority of such studies have found IFOF stimulation to cause semantic paraphasias (Duffau et al., 2003, 2008, Duffau et al., 2009; Mandonnet et al., 2007; Gil-Robles et al., 2013; Moritz-Gasser et al., 2013; Motomura et al., 2014, 2018; Almairac et al., 2015; Duffau, 2015; Fernández et al., 2020). ILF’s role is more contested; some have found ILF stimulation to cause omission errors (Herbet et al., 2019) and some have found no adverse effect on language production when stimulating this tract (e.g., Mandonnet et al., 2007; Gil-Robles et al., 2013; Moritz-Gasser et al., 2013). Collectively, the body of literature reviewed above supports the involvement of these tracts in semantic-lexical processing, with a suggestion that the ILF may be more strongly involved in lexical-to-semantic mapping (i.e., comprehension) and IFOF in semantic-to-lexical mapping (i.e., production). This distinction was supported in a study by Harvey and Schnur (2015). There are, however, other studies that have attributed comprehension vs. production functions to different parts of each tract. For example, some studies have claimed that the middle and posterior parts of the ILF and IFOF are related to comprehension (Ivanova et al., 2016; Zhang et al., 2018), whereas anterior portions of these tracts are important for speech production (Ivanova et al., 2016; Tuncer et al., 2021). Focusing on disentangling the comprehension/production contribution of ILF and IFOF is a fruitful area for future research.

More recent studies have started to tap more closely into the functions of ILF and IFOF in terms of semantic-lexical *control*. Control has been discussed in two senses in the language literature: conflict and weak associations (e.g., Thompson-Schill and Botvinick, 2006; Martin-Chang and Levy, 2006; see Nozari et al., 2016a for a unifying view). For example, words belonging to the same semantic category but with opposite valences can make semantic judgment harder by inducing conflict. Similarly, words with distant relationships are harder to judge as related, because of the weak association between them. Dávolos et al. (2020) presented participants with visual cue words, followed by three words, from which to choose the most related word to the cue word. Congruency and strength of association were manipulated between the cue and target words and were shown to be correlated with FA in the left ILF, suggesting a role of this tract in lexical-semantic control. FA in the right IFOF, on the other hand, was related to processing under low demands (i.e., no conflict, strong association). In another study, Harvey and Schnur (2015) compared semantic interference in production (using the blocked cyclic naming task) and comprehension (using a word-to-picture matching task) in 18 participants with left hemisphere stroke. They reported an association between the ILF and semantic interference in word comprehension, whereas the IFOF was critical for resolving semantic interference in production. Finally, Nugiel et al. (2016) used a verb generation task in which verbs were elicited from neurotypical individuals either by nouns that strongly elicit a certain verb (e.g., scissors → cut) or by nouns that are loosely associated with multiple verbs (e.g., ball → throw, roll, play, etc.), with the latter requiring greater semantic-lexical control. They found both ILF and IFOF’s structure to be predictive of semantic control in this study. This brief review shows that there is growing evidence for the critical contribution of these two tracts not just to semantic retrieval, but specifically to semantic control, although it remains debated whether the tracts contribute differently to semantic control in comprehension vs. production.

Lastly, we must also mention verbal fluency, as a test commonly used in assessing the contribution of white matter to language production. Verbal fluency tasks come in two varieties: semantic or category fluency refers to a test in which participants are prompted to produce as many words as possible from a certain semantic category (e.g., animals). Phonemic or letter fluency tasks, on the other hand, require participants to produce as many words beginning with a certain sound or letter, irrespective of their semantic category. The idea is that the two variants index semantic-lexical and lexical-segmental processing, respectively. One issue with verbal fluency tasks is that they confound primary production functions (i.e., the ability to activate, retrieve, and produce a word) with cognitive control functions that are often inherent to these tasks. For example, scoring high on the category fluency task when the target group is “animals” often entails a strategy of focusing on a sub-category (e.g., farm animals), exhausting that, and then successfully switching to a new sub-category (e.g., sea animals; Hirshorn and Thompson-Schill, 2006). This ability requires cognitive control. Without detailed analyses to extract the processes involved in this task, it is unclear whether a correlation between performance and white matter measurements is reflecting a primary production operation or, instead, the implementation of control in language production. This caveat makes the interpretation of findings from these tasks difficult, as will be seen in several of the following sections in which we review the results of correlating performance in verbal fluency tasks with the properties of different tracts. For example, several studies have found a correlation between ILF, IFOF, or both in category fluency tasks (e.g., Almairac et al., 2015; Griffis et al., 2017; Gonzalez et al., 2021), but there are also reports of an association of the same tracts with phonemic fluency performance (e.g., Sanvito et al., 2020). Rather than pointing to the involvement of ILF and IFOF in both semantic and phonological processing, these findings most likely show the underspecified nature of the verbal fluency tasks for pinpointing cognitive functions.

### 5.4. Summary

In addition to visually guided tasks, including reading, the evidence suggests that ILF and IFOF play a clear role in semantic-lexical processing in language. The evidence seems to point to a more prominent role of the ILF in comprehension and the IFOF in production, although alternative accounts, e.g., a posterior-anterior division of labor in each tract, have not been conclusively ruled out. Finally, there is clear evidence linking IFOF and ILF to semantic-lexical control, although more studies will be useful to examine whether a division along comprehension/production lines also exists in the control functions of these tracts.

## 6. Uncinate fasciculus (UF)

### 6.1. Anatomy

**Figure 3:**
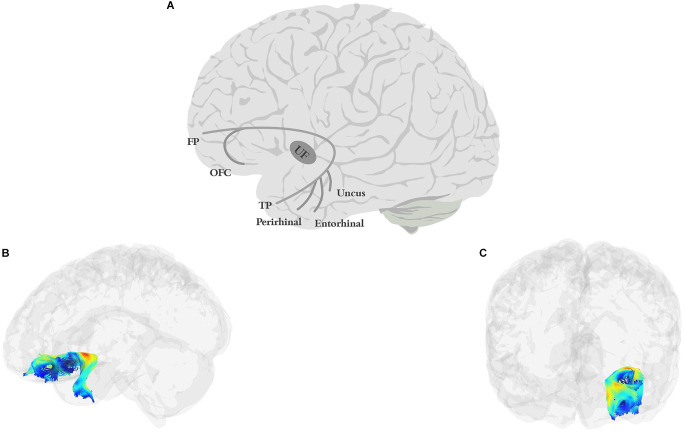
Anatomy of UF (**A**: schematic; **B**: tractography, sagittal view; **C**: tractography, coronal view). FP, frontal pole; OFC, orbitofrontal cortex; TP, temporal pole.

The UF (Figure 3) is a short, hook-shaped bidirectional fiber bundle around the Sylvian fissure, running through the extreme and external capsule, and connecting the temporal pole with the orbitofrontal cortex (OFC; Horel and Misantone, 1976; Ebeling and Cramon, 1992; Kier et al., 2004; Von Der Heide et al., 2013; Papinutto et al., 2016; Briggs et al., 2018a). The temporal segment originates from the uncus, entorhinal, and perirhinal cortices, and temporal pole/anterior temporal lobe (Ebeling and Cramon, 1992; Von Der Heide et al., 2013). The frontal termination of the UF has two branches: a larger ventrolateral branch and a smaller medial branch. The ventral branch terminates in the lateral orbitofrontal cortex while the medial branch terminates in the frontal pole (BA 10; Von Der Heide et al., 2013).

### 6.2. Function

Given the essential role of the orbitofrontal cortex and the limbic system in social cognition (e.g., Rushworth et al., 2007) and the extensive connections between the UF and these regions, it is not surprising that abnormalities of the UF have been frequently observed in autism spectrum disorder (ASD; Pugliese et al., 2009; Ameis et al., 2011; Lo et al., 2011; Poustka et al., 2012; Travers et al., 2012), conduct disorder (CD; Passamonti et al., 2012; Sarkar et al., 2013; Zhang et al., 2014), social anxiety disorder (Phan et al., 2009; Tröstl et al., 2011; Baur et al., 2012), and schizophrenia (Burns et al., 2003; Kubicki et al., 2005; Kitis et al., 2012; Jung et al., 2020). Also, several studies have demonstrated a correlation between the UF and emotional processing, including the interpretation of emotions and the expression of empathy (Zuurbier et al., 2013; Oishi et al., 2015; Nakajima et al., 2018; Coad et al., 2020; Granger et al., 2021). The connection established between the anterior temporal lobe and orbito-frontal cortex and the bidirectional flow of information in the UF has also led researchers to propose that the tract is heavily involved in modulating mnemonic representations in the temporal lobe through a temporo-frontal reward-punishment loop, i.e., a learning reinforcement loop for forming episodic memory and learning (see Von Der Heide et al., 2013, for a review). Compatible with such a position, the UF has been implicated in learning abilities in both verbal and non-verbal tasks (Thomas et al., 2015; Alm et al., 2016; Rossi et al., 2017), and performance in memory tasks, especially, but not exclusively, verbal memory (Diehl et al., 2008; Fujie et al., 2008; Serra et al., 2012; Christidi et al., 2014; Schaeffer et al., 2014). In terms of lateralization, some studies have reported left-lateralized functions (Kubicki et al., 2002; Hervé et al., 2006; Rodrigo et al., 2007; Hasan et al., 2009), while others have reported right-lateralized functions (Highley et al., 2002; Park et al., 2004), which may be explained, at least in part, by different roles of the tract in cognitive and emotional processing.

### 6.3. Links to language

This tract’s connection to the ATL also brings up the possibility of involvement in semantic processing. Generally aligned with a role in semantic processing, UF deficits are often observed in dementia, including the semantic variant of PPA (Agosta et al., 2013; Powers et al., 2013; Iaccarino et al., 2015; Tu et al., 2016; Briggs et al., 2018a; Bouchard et al., 2019), with some studies claiming decreased FA of the UF to be the main predictor of semantic dementia (Agosta et al., 2012; Bouchard et al., 2019), and others showing a correlation between cognitive decline and the integrity of the UF (Morikawa et al., 2010; Hiyoshi-Taniguchi et al., 2015). Moreover, the UF has been implicated, along with IFOF, in several studies that have used a battery to tap into semantic processing (de Zubicaray et al., 2011; Mirman et al., 2015; see also semantic processing deficits in schizophrenia; Surbeck et al., 2020). If the UF is involved in semantic selection, one could naturally expect it to be important for a variety of language functions that involve semantic processing. In keeping with this, the UF is also often implicated in language and language disorders. For example, in stroke patients, some studies have reported UF’s FA values to correlate positively with various measures of language processing, including auditory comprehension, naming, and spontaneous speech (Fridriksson et al., 2013; Zhang et al., 2021; cf., Ivanova et al., 2016).

In PPA too, UF’s integrity has been linked to naming and category fluency performance (Catani et al., 2013; Powers et al., 2013). Similarly, the microstructure of the UF is predictive of core language scores in children (Dodson et al., 2018). Finally, several studies have implicated the UF in reading abilities, including phonemic decoding efficiency (Bakhtiari et al., 2014; Welcome and Joanisse, 2014; Cummine et al., 2015; Arrington et al., 2017). Evidence directly linking the UF to picture naming is mixed. Some studies have reported that intraoperative stimulation of the UF causes lexical and semantic paraphasia, hesitations, and omission errors in picture naming (Raffa et al., 2016). Others have reported that simple language production tasks like picture naming and counting were not disturbed by the UF stimulation (Duffau et al., 2009). Finally, Jarret et al. (2022) suggested that UF (along with the ILF) constituted an indirect pathway for picture naming.

One possibility is that of a more nuanced picture. Recall that the UF connects ATL to the prefrontal cortex. It is thus possible that its role is not simple semantic retrieval, but semantic control (which is achieved through the interaction between temporal and frontal cortices; e.g., Thompson-Schill et al., 1997). If this is true, one would expect a critical contribution of the UF to tasks with greater control demands on the semantic-lexical system. Specific tests of the role of the UF in semantic-lexical control in tightly controlled studies are rare in the literature, and the current evidence is mixed. Harvey et al. (2013) tested 10 stroke survivors on two tasks that require semantic/semantic-lexical control, Pyramids and Palm Trees (PPTT), and an auditory word-to-picture matching task with semantic, phonological, and unrelated lures. After controlling for total lesion volume, the integrity of UF was a significant predictor of performance both on PPTT and on the word-to-picture matching task with semantic lures, suggesting a role of the UF in semantic/lexical control. Interestingly, this study found no relationship between semantic control and either ILF or IFOF (cf., Harvey and Schnur, 2015). In contrast, Nugiel et al. (2016) found no relationship between performance in the verb generation task, which as explained in the previous section indexes semantic-lexical control when multiple competitors are equally activated, and the microstructural measures of UF, whereas both ILF and IFOF were implicated in that study.

The UF is also frequently linked to performance in semantic fluency tasks in various populations, including individuals with Parkinson and Alzheimer’s disease (Lauro et al., 2010; Papagno et al., 2011, 2016; Rodríguez-Aranda et al., 2016; Di Tella et al., 2020), but as explained in the previous section, it is hard to disentangle semantic retrieval from semantic control in the category fluency tasks. Moreover, several studies have suggested UF’s involvement in phonemic/letter fluency tasks (Papagno et al., 2011; Serra et al., 2012; Kljajevic et al., 2016), even though some have reported greater contribution to semantic over letter fluency performance (Li et al., 2017). If phonemic/letter fluency truly indexes segmental processing abilities, this finding, together with the role of the UF in phonemic decoding proposed in reading, may attribute a dorsal-stream function to a ventral tract, a conclusion that does not fit well with the rest of this tract’s function, or with the null effects regarding its influence on segmental processing (e.g., no phonological errors or disruption in phonological processing during its stimulation; Duffau et al., 2009; Nomura et al., 2013; Raffa et al., 2016). A more plausible explanation is that the phonemic/letter fluency tasks also tap into other abilities, e.g., controlled lexical selection, that are more aligned with the functions of the UF. This can be tested by ruling out UF’s function in tasks that require phoneme/letter processing without strong demands on semantic-lexical selection.

One last finding that is relevant here is the selective impairment of naming pictures of famous people and objects when the UF is removed: in a study of 44 glioma patients before and after the surgical removal of UF, Papagno et al. (2011) reported changes to word production in picture naming, naming of famous faces, as well as impaired performance on verbal fluency tasks. However, a later study that followed up on 17 glioma patients up to 9 months after their surgery, showed that performance on picture naming and verbal fluency tasks had been restored to normal, but patients still had a significant impairment in the famous-face naming task (Papagno et al., 2016). It is possible that this selective impairment is linked to emotional processing (Papagno et al., 2016) or to semantic control and selection, both of which are more marked for famous faces and places than for generic objects.

### 6.4. Summary

UF’s connections to the orbito-frontal cortex imply a clear role for this region in processing reward and punishment, which is connected to both social/emotional processing and reward-based learning. These functions can affect various aspects of language processing, especially those with a social component. The question is whether, beyond these, the linking of ATL to the prefrontal cortex implies a role for the UF in semantic-lexical selection in situations with high selection demands. Current results sometimes implicate the UF, sometimes ILF and/or IFOF, and sometimes a combination of these tracts. One interpretation of these diverging results is that the UF plays a complementary role to ILF and IFOF in semantic processing (Cocquyt et al., 2020). More empirical data will be helpful in testing this hypothesis.

## 7. Extreme capsule (EmC)

### 7.1. Anatomy

**Figure 4:**
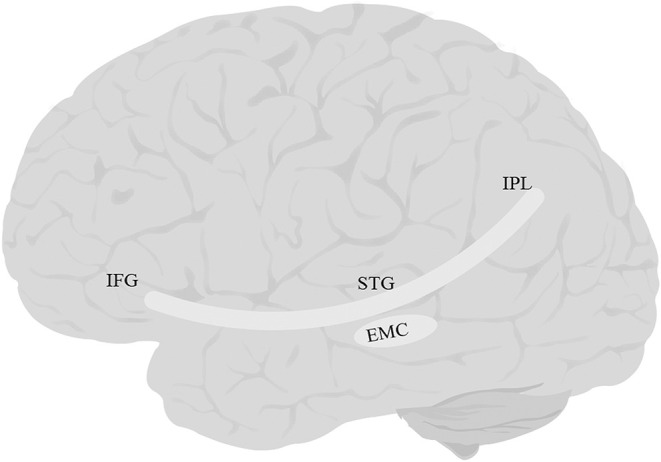
Anatomy of EmC. EmC, extreme capsule; IFG, inferior frontal gyrus; IPL, inferior parietal lobe; STG, superior temporal gyrus. The fibers of this tract overlap with IFOF, and UF, and therefore tractography of this tract was not possible.

The EmC (Figure 4) is often mentioned in studies of white matter. However, researchers vary greatly in their definition of what the EmC is. Some view it simply as a topographical region between the insula and claustrum (Axer et al., 2013). Others have described it as part of the IFOF or UF or the MdLF/ILF pathway (Saur et al., 2008; Northam et al., 2012; Patterson et al., 2014; Verly et al., 2019). Yet others have described it as a more substantial fiber complex or system, e.g., the “extreme capsule fiber complex” (Mars et al., 2016) or the “extreme capsule fascicle” (Martinez Oeckel et al., 2021), and have included large sections of the ventral pathway in this bundle. Finally, in a careful study focused specifically on delineating the trajectory of EmC, Makris and Pandya (2008) were able to clearly distinguish between the tract and the adjacent MdLF, UF, AF, and SLF-II, and SLF-III. They defined EmC as a long, left-lateralized association fiber coursing between the inferior frontal cortex and the STG, extending into the inferior parietal lobule.

### 7.2. Function and links to language

Given the temporal origin of the tract which is close to Heschel’s gyrus, auditory processing has been one of the proposed functions of this tract (Frey et al., 2008). More nuanced hypotheses have been formed around the connection that this tract provides between the STG and IFG, in terms of mediating verbal working memory. For example, Lopez-Barroso et al. (2011) showed that performance under articulatory suppression (asking participants to repeatedly produce the syllable “bla” while listening to the language stream) was significantly correlated with FA in left EmC and the external capsule. Naturally, a role in verbal working memory links EmC to language learning. In line with this hypothesis, Wong et al. (2011) trained participants in a sound-to-word learning paradigm, where they had to use foreign phonetic contrasts to access meaning. They found the FA of a left temporo-parietal region to be correlated with learning in this paradigm, and reported EmC, along with ILF, to mediate auditory comprehension.

Studying 25 individuals with aphasia and 24 healthy controls, Kourtidou et al. (2021) found that radial diffusivity of the right temporo-frontal extreme capsule fasciculus was predictive of a number of language functions, including oral and reading comprehension, word and sentence repetition, and number of words/minute produced in storytelling tasks, such as the Cookie Theft Picture Test (see also Martinez Oeckel et al., 2021). Others have proposed EmC’s involvement in various language functions including speech rate in stroke survivors (Efthymiopoulou et al., 2017), semantic paraphasia in glioma patients in intra-operative stimulation (Duffau et al., 2005), comprehension (Kümmerer et al., 2013), picture naming (Jarret et al., 2022), and vocabulary development in children with developmental language disorders (Verly et al., 2019), while others have reported no relationship between EmC’s lesion size and language production abilities such as naming and rate and informativeness of speech (e.g., Marchina et al., 2011). Finally, impaired syntactic processing has also been linked to EmC deficits in some studies (e.g., Papoutsi et al., 2011; Rolheiser et al., 2011; Griffiths et al., 2012), but not in others (Wilson et al., 2011; Teichmann et al., 2015).

### 7.3. Summary

To summarize, EmC’s function is not well understood, partly due to the lack of consensus about its anatomical definition, but there is more and more evidence that the tract represents a pathway distinct from its neighboring white matter, and may be involved in some aspect of language processing. So far, detailed studies of the linguistic function of the EmC have been relatively sparse, but the connection between STG and frontal cortex, and its implication for sound processing and verbal working memory, appears to be a promising venue for more theoretical approaches to the possible function of this tract in language acquisition, comprehension, and production.

## 8. Middle longitudinal fasciculus (MdLF)

### 8.1. Anatomy

**Figure 5:**
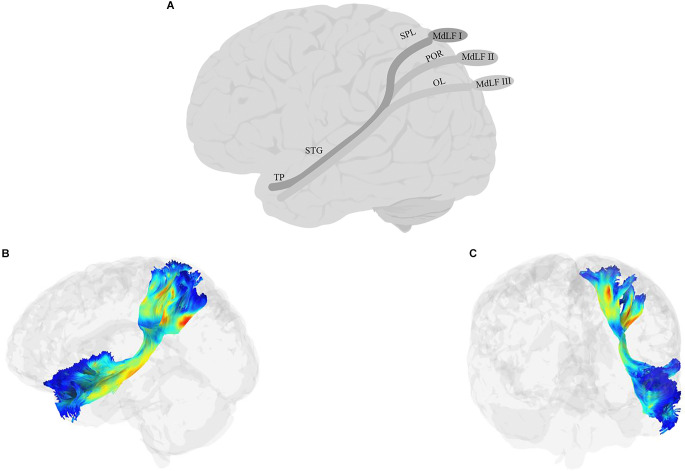
Anatomy of MdLF (**A**: schematic; **B**: tractography, sagittal view; **C**: tractography, coronal view). OL, occipital lobe; POR, parieto-occipital regions; TP, temporal pole.

The MdLF (Figure 5) is a long association fiber that connects temporal regions with parietal and occipital lobes (Burks et al., 2017; Conner et al., 2018b). First reported by Seltzer and Pandya (1984) using autoradiographic histological tract-tracing and later confirmed using more recent non-human tract-tracing studies (Schmahmann et al., 2007), the pathway had been historically absent from human anatomical reports (Burdach, 1826; Foville, 1844; Meynert, 1885; Dejerine, 1895) and even some recent anatomical atlases (e.g., Oishi et al., 2010) and studies of white matter tracts (Catani et al., 2005; Bürgel et al., 2006; Wakana et al., 2007; Catani and Thiebaut de Schotten, 2008; Hua et al., 2008; Holl et al., 2011; Thiebaut de Schotten et al., 2011). More recent studies in humans, however, have begun to identify MdLF as a distinct pathway. Some, such as Saur et al. (2008) discuss the MdLF as two composite fiber bundles, one in the dorsal pathway together with AF/SLF and one in the ventral pathway together with the ILF. Others have identified the MdLF as an independent tract extending from the AG to the anterior superior temporal cortex, running dorsal and medial to the AF/SLF (Frey et al., 2008; Makris and Pandya, 2008; Turken and Dronkers, 2011; Wong et al., 2011; Menjot de Champfleur et al., 2013). Today, researchers agree that the tract is heavily involved in connecting STG to other parts of the cortex, although there is not always consensus among studies on what these other parts are. Candidates include other regions in the temporal cortex, such as MTG (Turken and Dronkers, 2011), parietal regions such as the superior parietal lobule/precuneus and AG (Turken and Dronkers, 2011; Wang et al., 2013; Makris et al., 2013), and possibly some of the occipital regions such as the cuneus, and lateral occipital lobe (Makris et al., 2017). In one of the most recent attempts to define the anatomical branches of the MdLF, Kalyvas et al. (2020) performed a combined study of cadaveric dissections together with DTI in neurotypical adult participants and identified three branches of the MdLF (Figure 5): the first, MdLF-I, connects TP and STG to the SPL through Heschel’s gyrus. The second, MdLF-II, connects TP and STG to the parieto-occipital regions. The third, MdLF-III connects the most anterior part of TP to the posterior part of the occipital lobe through the AG.

### 8.2. Function and links to language

Based on its clear connections to the temporal lobe and AG, there have been attempts to link MdLF to language processing. For example, by bundling the AF/SLF with one branch of the MdLF and ILF with another branch of MdLF, Saur et al. (2008) proposed the former’s role in sublexical repetition and the latter’s role in comprehension in 33 neurotypical adults. However, because of bundling with other fibers in the dorsal and lateral pathways, respectively, it is difficult to isolate the role of MdLF in such tasks. MdLF has not been a frequent target of aphasia studies, but the little evidence that exists is mixed. In a study of 20 individuals with PPA, Luo et al. (2020) reported significant correlations between word comprehension and naming and the white matter changes to the MdLF in the dominant hemisphere. In contrast, Blom-Smink et al. (2020) found no clear link between the integrity of MdLF and naming performance in 10 individuals with sub-acute post-stroke aphasia. Finally, in one of the few intraoperative electrostimulation studies investigating the role of MdLF in language, De Witt Hamer et al. (2011) tested counting and picture naming of eight glioma patients after the stimulation of MdLF and found no changes to either task. Moreover, the resection of the left MdLF did not result in impaired naming. It must be noted, however, that resection was not complete in all patients, and only included the part of the MdLF anterior to Heschel’s gyrus, therefore, these results must be interpreted with caution.

The brief review above highlights the sparsity of research on the role of MdLF in language processing, but the few existing results do not seem to provide strong support for an essential role of this tract at least in language production, including the semantic-lexical mapping required for picture naming. Two findings in the anatomical study of Kalyvas et al. (2020) provide theoretical support for the non-critical role of MdLF in language processing: (1) there is no clear termination of the fibers from any of the MdLF branches in the AG, and (2) No clear leftward symmetry of the tract (also see Makris and Pandya, 2008; Makris et al., 2013, 2017; Kamali et al., 2014 for differences in the asymmetry index). In contrast, the centrality of STG and the auditory cortex in at least two branches of this tract motivates a role in auditory processing (Saur et al., 2010; Dick and Tremblay, 2012). Evidence in support of this view is more convincing, although there is much room for additional evidence and careful studies. For example, MdLF was one of the ventral pathways implicated in the study of Wong et al. (2011) where they measured participants’ ability to learn new phonetic contrasts for discriminating words in a foreign language. But perhaps the most detailed study of the role of MdLF in auditory processing is the study of Tremblay et al. (2019), in which the authors used high angular resolution diffusion imaging (HARDI) with advanced anatomically constrained particle filtering tractography algorithms that are robust against problems such as crossing fibers and partial volume effects, to disentangle the role of AF and MdLF in auditory processing. Younger and older adults participated in a syllable discrimination task with broadband masking noise. After controlling for differences in individuals’ hearing sensitivity, an age-independent effect linked both tracts to performance in the task, but in relatively distinct ways: while AF was predictive of sensitivity (d-prime in the signal detection framework), the MdLF was linked to response bias (criterion in the signal detection framework). These results suggest a distinct role for MdLF in higher-level auditory processing, such as decision making.

### 8.3. Summary

The MdLF is a relatively understudied tract. But recent evidence suggests that it is a distinct tract in humans and that it has possibly up to three separate branches. Of the roles proposed so far for this tract, an involvement in auditory processing is the most plausible and well-supported role. The nature of such involvement, however, remains underspecified. Future studies should clarify the extent to which the processing is speech-specific (or not), and whether the tract’s role is more pronounced in cognitive aspects of auditory processing, such as implicit or explicit decision making. Finally, the links to the occipital cortex remain intriguing, and potentially related to processes mediating audiovisual integration (Wang et al., 2013), although an empirical investigation of this hypothesis has, to our knowledge, not yet been carried out.

## 9. Superior longitudinal fasciculus (SLF)

### 9.1. Anatomy

When we think about the classic language pathway, we often think about Geschwind’s iconic illustration of a pathway connecting Broca’s and Wernicke’s areas. For years, variations of this pathway connecting the inferior frontal cortex with temporal and parietal lobes comprised a non-dissociable SLF/AF bundle, which has been called by various names, including the Burdach fasciculus, the superior longitudinal bundle or the arcuate bundle (e.g., Burdach, 1826; Dejerine, 1895; Wernicke et al., 1897). More recently, the two tracts have been deemed distinct, although AF is still widely considered as one of the branches of the SLF.

**Figure 6:**
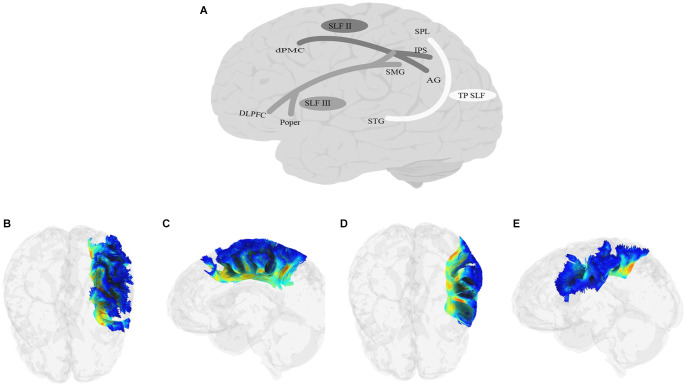
Anatomy of SLF (**A**: schematic; **B**: tractography, axial view of the SLF II; **C**: tractography, sagittal view of the SLF II; **D**: tractography, axial view of the SLF III; **E**: tractography, sagittal view of the SLF III; SLF-I is not shown due to lack of relevance to language processing). DLPFC, dorsolateral prefrontal cortex; dPMC, dorsal premotor cortex; IPS, intraparietal sulcus; Poper, Pars opercularis; SPL, superior parietal lobule; TP, temporoparietal.

SLF (Figure 6) is a bundle of association fibers that connects the superior and inferior parietal cortices to the frontal cortex (Petrides and Pandya, 1984; Yeterian et al., 2012). The SLF is usually divided into three distinct branches, SLF-I, SLF-II, and SLF-III (Petrides and Pandya, 1984, 2002, Petrides and Pandya, 2006; Yeterian et al., 2012; Caverzasi et al., 2016; Barbeau et al., 2020), although some disagreement remains about the exact origin and destination of each branch. SLF-I is the dorsal-most branch, and connects the superior parietal lobule and precuneus to the superior frontal cortex, the dorsal premotor area, the SMA, and possibly the anterior cingulate cortex (Petrides and Pandya, 1984; Schmahmann and Pandya, 2006; Schmahmann et al., 2007; Thiebaut de Schotten et al., 2012; Yeterian et al., 2012). SLF-II originates in the caudal inferior parietal lobule, the intraparietal sulcus, and the angular gyrus and terminates in the DLPFC, including the dorsal premotor area (Petrides and Pandya, 1984, 2002, Petrides and Pandya, 2006; Yeterian et al., 2012). The SLF-III is the ventral-most branch and connects the rostral part of the inferior parietal lobule, i.e., the supramarginal gyrus, and the anterior parts of the intraparietal sulcus to the ventral premotor cortex and the caudal banks of the arcuate and principal sulci (Petrides and Pandya, 1984, 2002, Petrides and Pandya, 2006; Yeterian et al., 2012). Recently, Barbeau et al. (2020) proposed a division of SLF-III into two branches, the ventral branch terminating in BA 6 (pre-SMA and SMA) and BA 44 (pars opercularis of IFG), and the dorsal branch terminating in BA 9 and BA 46 (DLPFC). Finally, some studies describe a temporoparietal component of the SLF, which traverses from the posterior part of the STG to the inferior and superior parietal lobules, and is often labeled SLF-tp (e.g., Caverzasi et al., 2016), and is sometimes further divided into SLF-tp-IPL (inferior parietal lobule) and tp-SPL (superior parietal lobule; Kamali et al., 2014; Bullock et al., 2019). It is worth mentioning that AF, which courses in parallel to SLF-III and connects the temporoparietal junction to the frontal cortex, has sometimes been considered a part of the larger SLF bundle. In this article, we will discuss AF separately in the next section.

### 9.2. Function

Empirical studies do not always separate the three branches of the SLF, but in those which do, it is often the SLF-III and sometimes the SLF-II that are related to language processing. Due to its anatomical location and connections, SLF-I is usually not considered a language-related tract. Its main connectivity is to the superior parietal cortex, which encodes body part locations in relation to space and eye-hand coordination for reaching. Consequently, damage to the caudal part of the superior parietal lobule can cause optic ataxia, i.e., difficulty in visually guided reach (Naito et al., 2008; Ferraina et al., 2009; Granek et al., 2012). By connecting this region to the premotor cortex, SLF-I is thought to play a role in regulating fine motor behavior, especially in tasks that require selection among competing motor plans (Hyde et al., 2021). SLF-tp is not always mentioned, but together with AF, there are reports of its connection to the language (Caverzasi et al., 2016). Galantucci et al. (2011) claimed to have separated SLF-tp from the temporoparietal AF and showed that only damage to the former was observed in the logopenic variant of PPA. They also reported SLF-tp’s damage in the nonfluent variant of PPA.

### 9.3. Links to language

The investigation of the linguistic functions of the SLF has ranged from broad to specific aspects of language processing. For example, in a study of 20 children between 8 and 10 years of age, Asaridou et al. (2017) showed that children’s vocabulary growth was uniquely predicted by the cortical thickness of the left SMG, and concluded that the direct link between this region and the IFG provided by SLF-III makes the tract a critical pathway for the development of the lexicon. Likewise, in a comparison between children on the autism spectrum with and without language impairment, Nagae et al. (2012) linked elevated mean diffusivity values of SLF to language impairments. More recently, Gao et al. (2020) showed increased FA in the right SLF in bilingual compared to monolingual speakers (but see Wang et al., 2016, for reports of left-lateralization of SLF-II and SLF-III). The link is stronger for production than comprehension. For example, in a study of 49 typically developing children and adolescents ranging from 5–17 years of age, Urger et al. (2015) reported that only production but not comprehension was associated with the FA of left SLF. Likewise, Hillis et al. (2018) reported a link between naming recovery in post-stroke aphasia and the integrity of SLF/AF.

More detailed hypotheses usually concern the role of SLF-III: by the virtue of connecting the SMG, which is frequently implicated in phonological processing and phonological deficits (e.g., Schwartz et al., 2012), to the frontal cortex, this branch of SLF is a prime candidate for language processing, especially phonological production. The more recent division into ventral and dorsal branches terminating in premotor and DLPFC, respectively (Barbeau et al., 2020), has further motivated finer-grained distinctions in the function of this tract into mapping phonological codes onto abstract motor plans (Bohland et al., 2010; Miller and Guenther, 2021) vs. maintaining phonological information in working memory. In keeping with this hypothesized role, several lines of research have linked phonological processing abilities to SLF (and in some cases, specifically to SLF-III). First, measures of phonological awareness are often correlated with the properties of SLF. The tests used to measure phonological awareness vary, but often include tasks such as sound matching, elision, and word blending. In sound matching tasks, participants hear two or more stimuli and must make a perceptual judgment about whether they match or not. Elision tasks measure the ability to remove phonemes from spoken words to form other words, and blending words tasks measure the ability to synthesize phonemes to form words. In a comparison of two groups of children born pre-term and full-term, tested when 6 years old, Dodson et al. (2018) showed a significant association between the FA of the left SLF (and AF) and phonological awareness measures (see also Travis et al., 2017).

A second phenomenon linking SLF to phonological abilities in production is the Tip of the Tongue (TOT) state. This is a state in which the speaker almost remembers a word but cannot fully produce it. TOT states usually benefit from phonological cues more than semantic cues, which has led to the localization of the problem to the mapping of lexical items to phonemes (Meyer and Bock, 1992). Stamatakis et al. (2011) tested 24 neurotypical adults between 19 and 82 years of age on the Boston Naming Task and showed that even though accuracy was associated with several tracts, including SLF, the TOT state was uniquely related to the most posterior part of the left SLF and a homologous area in the right hemisphere (cf., Kljajevic and Erramuzpe, 2019). A third line of research linking SLF to phonological abilities is the neuropsychological data on word production. McKinnon et al. (2018) tested 32 participants with chronic post-stroke aphasia and showed that the probability of making semantic and phonological errors was strongly linked to the ILF and SLF axonal loss, respectively (see also Han et al., 2016; Kyeong et al., 2019; Stark et al., 2019). Similarly, in a study of 24 participants with PPA, Powers et al. (2013) showed that participants’ naming performance in the logopenic—but not the semantic—variant was associated with the integrity of the left SLF. Moreover, auditory repetition performance, which has a stronger emphasis on phonological encoding than lexical retrieval (Nozari et al., 2010; Dell et al., 2013; Nozari and Dell, 2013), has also been linked to the SLF (Breier et al., 2008). Adding to this evidence are data from individuals who stutter. Chang et al. (2015) studied 77 children who stuttered and reported that these children, compared to controls, showed decreased FA of the left SLF and the gray matter regions connected by this tract, including IFG, premotor, motor, STG, MTG, and inferior parietal regions. Likewise, intraoperative stimulation of SLF has led to dysarthria and anarthria (Maldonado et al., 2011), although distinguishing problems of articulation from phonological encoding is difficult, especially during surgery with limited time.

Finally, the last line of research connecting the SLF to phonological processing is the close link posited between the tract’s properties and reading abilities (e.g., Bakhtiari et al., 2014; Travis et al., 2017; Bruckert et al., 2019). SLF is one of the three major pathways related to the VWFA, providing critical connections between this region, STG, IFG, and DLPFC (Chen et al., 2019). Bruckert et al. (2019) showed that the FA values for the left and right SLF and left AF at 6 years of age were predictive of the oral reading index at age 8. These findings are mirrored in studies of dyslexia: Zhao et al. (2016) showed that, as a group, children with dyslexia demonstrated greater right-lateralization of SLF-II (together with less left-lateralization of IFOF) than non-dyslexic readers. The lateralization index was also predictive of reading performance in the dyslexic group (see also Hoeft et al., 2011). Even though reading requires more than just phonological processing abilities, such abilities do play an important role, especially in sublexical reading. Therefore, although not a sufficient piece of evidence by itself, the link between SLF and reading abilities adds to the other bodies of evidence connecting this tract to phonological processing abilities.

There are also many studies investigating the correlation between SLF properties and verbal fluency tasks. As noted in earlier sections, although verbal fluency is one of the most popular language tests in studies of the neurobiology of language, it is not nearly as pure of a measure for semantic and phonological abilities as it has often been assumed. Unsurprisingly, even though SLF has been implicated in both semantic and phonemic fluency tasks, these reports are not consistent across studies and populations (e.g., Powers et al., 2013; Madhavan et al., 2014; Pustina et al., 2014; Urger et al., 2015; Sanvito et al., 2020; Gonzalez et al., 2021), and in most cases do not differentially implicate SLF’s involvement in one task vs. the other. Rather than taking such evidence as suggesting a role for SLF in both semantic and phonological processing, it is plausible to consider the tract’s relevance to some general task demands shared by both phonemic and semantic verbal fluency tasks. For example, it has been suggested that the association between SLF and verbal fluency in studies of aging is in fact mediated by verbal working memory, which has been linked to the FA of the bilateral SLF (Peters et al., 2012).

The studies reviewed above clearly link SLF to phonological processing. More sporadic have been attempts to link SLF to syntactic processing. In a study of 17 neurotypical children, Mills et al. (2013) found that those with a more complex narrative had higher diffusion coefficients in their left SLF and (AF; see also Friederici et al., 2006 for implicating SLF in parsing sentences with complex hierarchical structures). A few neuropsychological studies have also provided support for the link between SLF and syntactic processing. Wilson et al. (2011) studied 27 participants with PPA and found reduced FA in the left SLF/AF to be correlated with syntactic deficits in both comprehension and production. In another PPA study, Marcotte et al. (2017) linked the deficits in the non-fluent variant (nfv) vs. the semantic variant (sv) to increased radial diffusivity in the left SLF vs. in the bilateral UF and ILF, respectively (see also Whitwell et al., 2010; Tetzloff et al., 2018). There is also a report of inducing grammatical gender errors produced in response to noun probe in nine French-speaking glioma patients undergoing surgery, as a result of what appears to be the stimulation of a part of SLF (Vidorreta et al., 2011).

Although not as clear as the motivation for linking SLF to phonological processing, it is possible that parts of the tract traversing from the parietal to the frontal cortex may connect the pMTG and IFG and thus carry out the syntactic operations proposed by Matchin and Hickok (2020) in production. More difficult is explaining the role of the tract in syntactic comprehension unless one notes the generally high demands on working memory and control processes often required in sentences with complex hierarchical structures that have often been used in experiments tapping into syntactic comprehension. In such cases, it is conceivable that the DLPFC and IFG’s connection to the temporoparietal regions may be essential for maintaining information actively in working memory and resolving competition between different interpretations. This is particularly important, as SLF, and especially SLF-II and SLF-III, have been independently implicated in working memory (Peters et al., 2012; Rizio and Diaz, 2016) as well as executive control (Urger et al., 2015; Ramsey et al., 2017; Linortner et al., 2020). Finally, unless carefully controlled in the experimental materials, syntactic complexity is often confounded with length, which itself imposes higher demands on phonological working memory, providing yet another alternative interpretation for the role of SLF in syntactic processing.

### 9.4. Summary

A rather large and converging body of evidence points to a clear role of the SLF (especially SLF-III, and possibly SLF-II) in phonological processing, especially in language production. SLF-tp is a promising but understudied candidate. Further studies can focus on disentangling the functions of the components of SLF, such as the maintenance of phonological information in working memory vs. mapping such information onto motor plans. There is also some evidence for the involvement of this tract in syntactic processing, but potential confounds such as higher demands on verbal working memory and executive control processes must be ruled out before the tract can be claimed to have a pure “syntactic” function. This is another great avenue for future research.

## 10. Arcuate fasciculus (AF)

### 10.1. Anatomy

**Figure 7:**
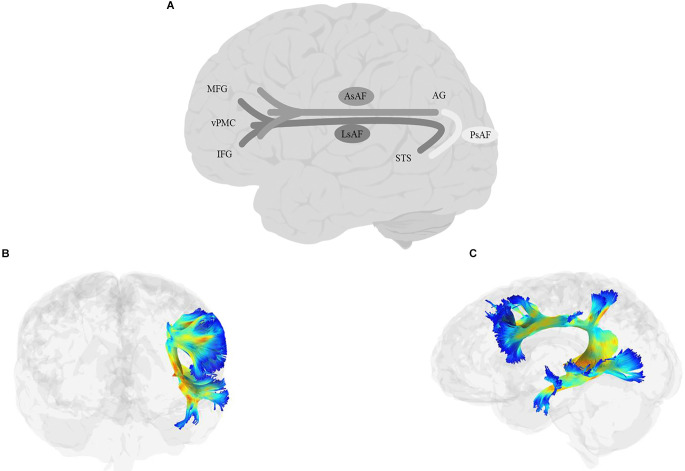
Anatomy of AF (**A**: schematic; **B**: tractography, coronal view; **C**: tractography, sagittal view). AG, angular gyrus; AsAF, anterior segment of the arcuate fasciculus; IFG, inferior frontal gyrus; LsAF, long segment of the arcuate fasciculus; MFG, middle frontal gyrus; PsAF, Posterior segment of the arcuate fasciculus; STS, superior temporal sulcus; vPMC, ventral Premotor Cortex.

The arcuate fasciculus (Figure 7) is a dorsal tract that connects the posterior superior temporal cortex (pSTC) to the IFG and ventral premotor cortex (vPMC; Catani et al., 2005, 2012; Thiebaut de Schotten et al., 2011; Weiner et al., 2017; Tremblay et al., 2019). Its anatomy is still under debate, and some studies suggested that the posterior part of the AF runs into the MTG and ITG (Rilling et al., 2008; Bernard et al., 2019; Giampiccolo and Duffau, 2022). Nevertheless, there is agreement that the tract has multiple branches (Dick and Tremblay, 2012; Dick et al., 2014). One common classification divides the tract into direct and indirect segments (Catani et al., 2005). The direct segment connects pSTC to IFG, MFG, and vPMC, while the indirect segment itself further divides into two components, a posterior component connecting STC to the inferior parts of the parietal lobe, and an anterior component connecting the inferior parietal areas to IFG, MFG, and vPMC (Catani et al., 2005, 2012; Thiebaut de Schotten et al., 2011; Weiner et al., 2017; Tremblay et al., 2019). A different model, the dual pathway architecture (Berwick et al., 2013; Brauer et al., 2013), also exists and is of potential functional importance. The key difference between the two branches in this model is not their origin (which is presumed to be pSTC in both cases), but rather their termination points, i.e., vPMC vs. IFG. It is hypothesized that the branch connecting pSTC and vPMC is involved in sensory-motor mapping and phonological processing, whereas the branch connecting pSTC to IFG is involved in higher-level processing, possibly syntactic processing. A critical difference between the AF in non-human primates and humans is that the tract is not strongly lateralized in monkeys (Eichert et al., 2019), but the evidence points to a prominent left-lateralization in humans (Vernooij et al., 2007; Lebel and Beaulieu, 2009; Sreedharan et al., 2015; Allendorfer et al., 2016; Silva and Citterio, 2017; Travis et al., 2017; Bruckert et al., 2019; Eichert et al., 2019; cf., Yeatman et al., 2011).

### 10.2. Function and links to language

The left-lateralization of AF in humans suggests a role of this tract in language processing. In line with this, the empirical evidence has often linked the left AF to language processing. For example, in a study of 106 neurotypical adults, Teubner-Rhodes et al. (2016) reported a positive correlation between vocabulary knowledge and microstructural properties of the left AF in the segment of the tract behind the posterior part of the Sylvian fissure. Similarly, the AF lesions have been linked to language deficits in aphasia (e.g., Tak and Jang, 2014; cf., Forkel et al., 2020), as well as language recovery (Primaßin et al., 2015). The critical role of the AF, irrespective of the damage to the gray matter, has been recently demonstrated in a study of 134 stroke survivors whose lesions were relatively limited to the left frontal cortex. Gajardo-Vidal et al. (2021) showed that production impairment lasting beyond 3 months after the stroke was selectively related to the damage of the anterior part of the AF directly above the insula, regardless of the damage to Broca’s area (see also Hillis et al., 2018). Interestingly, production scores were lower in the case of the AF damage with an intact Broca’s area compared to Broca’s damage with an intact AF, ruling out a central role for Broca’s damage in the impairment.

Some have claimed the involvement of the AF in both language production and comprehension (Turken and Dronkers, 2011). For example, Broce et al. (2015) showed that microstructural properties of this tract were predictive of both expressive and receptive language in children of 5–8 years of age. More recent studies have begun to localize these functions to different segments of the AF. For example, in a study of 33 individuals with aphasia, Ivanova et al. (2021) showed that AF’s anterior segment was predictive of fluency and naming, and its posterior segment of comprehension abilities. On the perception side, the role of the AF has been most extensively studied in auditory processing. Several studies have compared musicians and non-musicians in their ability to process fine-grained details of sound and have linked the superior performance in the former to different properties of the AF in that group (Oechslin et al., 2010; Moore et al., 2017; Li et al., 2021; cf. Perron et al., 2021). Interestingly, the volume and microstructural complexity of the tract also differ between vocal and instrumental musicians, compatible with the idea that the tract plays a special role in vocal-motor processing (Halwani et al., 2011). In non-musicians too, basic auditory processes have been linked to the microstructural properties of the AF (e.g., Vaquero et al., 2021). In a more detailed study, Tremblay et al. (2019) showed that sensitivity in a syllable discrimination task was related to the properties of the AF, while response bias was related to MdLF, putting AF in the center of the operations involved in fine-grained speech sound processing.

But the bulk of evidence, so far, is in favor of a role in production. In a study of 30 post-stroke individuals in the chronic phase, Marchina et al. (2011) showed that lesions to AF uniquely predicted naming ability, as well as the rate and informativeness of speech. In a study of 31 post-stroke individuals in the chronic phase, Halai et al. (2017) showed that speech fluency was uniquely correlated with the anterior section of the arcuate fasciculus. Several other studies in individuals with brain damage have also linked AF lesions to the fluency of spoken production (Fridriksson et al., 2013; Basilakos et al., 2014; Chenausky et al., 2020; Ivanova et al., 2021). Note that by “fluency” here, we mean fluency in the context of connected speech, as opposed to tests of “verbal fluency.” As alluded to in several of the earlier sections, the results of those tests are difficult to interpret. The issue is also evident in the AF literature: while some studies have reported a correlation between verbal fluency scores and the properties of AF (e.g., Blecher et al., 2019; Sanvito et al., 2020; Gonzalez et al., 2021), others have not (Phillips et al., 2011; Costentin et al., 2019). Moreover, the reports of AF lateralization are also not consistent across studies. Some have reported the involvement of the left (Blecher et al., 2019; Sanvito et al., 2020) and others of the right (Gonzalez et al., 2021) AF in letter fluency. Similarly, some studies have implicated the left (Blecher et al., 2019) and others of the right (Gonzalez et al., 2021) AF in semantic fluency. As for fluency in connected speech, there are many contributing factors (Nozari and Faroqi-Shah, 2017).

More evidence for the role of the AF in lower-level processing in production comes from its involvement in auditory repetition (Breier et al., 2008; Kim and Jang, 2013; Shinoura et al., 2013), especially sublexical repetition which indexes mapping input phonology to output phonology (Saur et al., 2008; Sierpowska et al., 2017). Similarly, damage to the AF has been shown to be specifically associated with phonological errors (Schwartz et al., 2012) as opposed to semantic errors, which arise at higher levels of processing in the production system (Nozari et al., 2011; Dell et al., 2014). This finding has been corroborated by the intraoperative stimulation of the AF or the AF termination sites, which has often led to speech errors (Giampiccolo et al., 2020), anomia (Duffau et al., 2002), and most pointedly, phonological errors (Mandonnet et al., 2007). Disentangling phonemic errors from dysarthric errors is not easy and has not been a focus in many of the studies reviewed above. It is thus possible that some of the problems reported in such studies are due to articulatory issues. In line with this idea, Liégeois et al. (2013) compared 32 individuals with a history of childhood TBI, and showed that those with persistent dysarthria had reduced FA in the left AF and reduced volume of the left AF and corpus callosum compared to those without dysarthria.

The critical role of the AF in phonological processing has also been tested with an array of phonological awareness tests. Different studies use different measures, and sometimes a mix of perception and production, but common tests include sound matching, elision, and word blending (see task definitions under the SLF section). The reports link the AF (often along with SLF, and often on the left side) to phonological awareness (Yeatman et al., 2011; Saygin et al., 2013; Gullick and Booth, 2014, 2015; Vandermosten et al., 2015; Dodson et al., 2018). Some researchers have also suggested that the auditory-motor mapping role of AF entails storing phonological information in working memory, essential for learning complex phonological sequences (Schulze et al., 2012). In keeping with this idea, López-Barroso et al. (2013) demonstrated a negative correlation between word learning and radial diffusivity of the long segment of the left AF in a group of adult participants learning an artificial language. Similar evidence followed in a longitudinal study of vocabulary development in children; Su et al. (2018b) followed the developmental trajectory of 79 children from age 4–14 years and reported a correlation between vocabulary development in the direct and posterior segments of the AF in the left hemisphere.

The AF has also been strongly linked to reading, at least in part due to its role in phonological processing. Thiebaut de Schotten et al. (2014) showed that increased FA and decreased perpendicular diffusivity of the temporoparietal portion of the left AF accompanied literacy. Furthermore, the microstructure of the AF was correlated with the response of VWFA to letter strings. In a longitudinal study of 30 children between the ages of 8 and 14 years, Gullick and Booth (2014) found a correlation between reading development and the FA of the direct segment of the AF for both younger and older halves of their sample (see also Yeatman et al., 2012; Gullick and Booth, 2015). The correlation between the AF and reading abilities has also been reported in children with dyslexia. In a longitudinal study of 75 children from ages 5–6–7–8 years, Vanderauwera et al. (2017) showed that only the left AF was exclusively related to the development of dyslexia (see also Hoeft et al., 2011). Also, a comparison of children with a family risk of dyslexia with a control sample showed that at-risk children had lower FA in the posterior AF (as well as the left IFOF; Vandermosten et al., 2015).

A special connection has also been suggested between the AF, as the tract connecting auditory to motor regions, and conduction aphasia, a deficit of mapping sensory to motor speech (Benson et al., 1973). For instance, individuals with conduction aphasia often show good semantic processing, but make phonological errors in naming. Importantly, their auditory repetition performance is markedly impaired compared to their naming performance. One suggestion has been that conduction aphasia may result from damage to the AF. There has been some empirical support for this idea, with lesions severely damaging AF and the surrounding tissue causing phonemic paraphasia and notable repetition deficits (e.g., Tanabe et al., 1987; Yamada et al., 2007). However, lesions to the AF do not necessarily cause conduction aphasia (e.g., Shuren et al., 1995; Selnes et al., 2002; Epstein-Peterson et al., 2012), suggesting that AF damage is not sufficient to produce this disorder.

Finally, some have attributed higher-level processing functions, such as syntactic processing, to AF. In one study, Mills et al. (2013) found a trend linking the syntactic complexity of the sentences produced by children to the diffusivity measures of the left AF. In another study, Papoutsi et al. (2011) found the AF lesions to be predictive of syntactic processing impairment. But it is important to note, as alluded to in the SLF section, that increasing syntactic complexity is often accompanied by increasing phonological complexity, i.e., longer phrases, more embeddings, etc. Therefore, unless specifically controlled for phonological processing load, drawing conclusions about the AF’s direct involvement in syntactic processing would be difficult.

### 10.3. Summary

The evidence reviewed above shows a clear role for AF in processing auditory details, mapping sound to motor actions, and more generally, phonological processing, especially in production. A possible role has also been proposed for syntactic processing, but so far, the evidence supporting this proposal has been limited. Moreover, careful controls for other factors that may increase processing load have not always been implemented in studies assessing the syntactic functions of AF. Disentangling phonological load from syntactic load would be a fruitful avenue for future studies on AF. Another fruitful avenue for future research would be to separate phonological and articulatory phonetic functions of AF (and SLF).

## 11. Frontal aslant tract (FAT)

### 11.1. Anatomy

**Figure 8:**
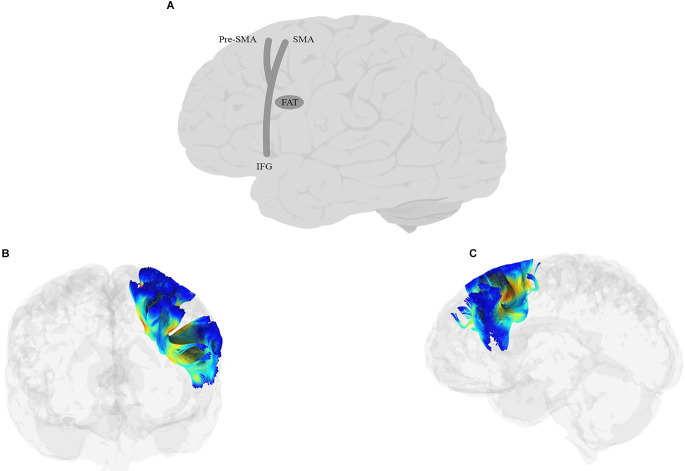
Anatomy of FAT (**A**: schematic; **B**: tractography, coronal view; **C**: tractography, sagittal view). IFG, inferior frontal gyrus; SMA, supplementary motor area.

The FAT (Figure 8) is a short association fiber connecting the lateral IFG to the SMA and pre-SMA in the superior frontal gyrus (Catani et al., 2012, 2013). Although a tract with these characteristics had been discussed before (e.g., Aron et al., 2007; Lawes et al., 2008; Oishi et al., 2008), the term “Aslant tract” was first coined by Catani et al. (2012); see also Thiebaut de Schotten et al. (2012), who described it specifically as a pathway connecting IFG’s pars opercularis to pre-SMA (see also Bozkurt et al., 2016). Today, the existence of this tract and its role in connecting IFG and the superior frontal gyrus is well established in both primates and humans (Petrides and Pandya, 2002; Martino and De Lucas, 2014; Briggs et al., 2018b).

### 11.2. Function and links to language

Based on its connectivity, several functions have been proposed for the role of FAT in cognition. Due to its clear connection to IFG, the debates that have impacted the role of IFG have directly impacted the ideas about the role of FAT. On the one hand, the classic view of Broca’s area as a key language region has generated the hypothesis that FAT’s connection to this region makes it a key language tract (e.g., Tremblay and Dick, 2016). The tract’s connection to pre-SMA and SMA and the link between language production and these regions has further supported FAT’s role in language processing (Tremblay and Gracco, 2009; Jarret et al., 2022). The alternative, domain-general view of IFG (e.g., Nozari et al., 2016a; cf. Nozari and Novick, 2017), on the other hand, has led some researchers to implicate FAT in conflict resolution functions, in both linguistic and non-linguistic domains, that have been attributed to this region (Dick et al., 2019). This view is further reinforced by the fact that pre-SMA and SMA have often been implicated in complex motor tasks, especially those that involve high competition (Derrfuss et al., 2004; Mars et al., 2007), such as the Flanker task (Ullsperger and von Cramon, 2001). Consequently, FAT’s links to these regions have been taken as a possible involvement of the tract in motor selection, conflict monitoring and resolution, and the execution of motor plans regardless of specific domains (e.g., Tremblay and Small, 2011). Dick et al. (2019) present a thorough review of the linguistic and non-linguistic functions of FAT, with an eye towards the tract’s laterality. Below, we briefly review the evidence and the conclusions.

The evidence linking the FAT to language processing and language disorders is ample. For example, a large-scale study of 834 participants from the Human Connectome Project found a significant correlation between bilateral FAT volume and language performance (Varriano et al., 2018). In another study, Broce et al. (2015) found that the length of the left FAT was predictive of language comprehension abilities in young children. Moreover, in stroke survivors, the extent of damage to FAT is predictive of the improvement of language skills (Sihvonen et al., 2021; c.f., Tuncer et al., 2021). Direct evidence for the importance of FAT in speech production comes from intraoperative stimulation of the tract, which often causes speech arrest (Vassal et al., 2014; Fujii et al., 2015; Kinoshita et al., 2015) or stuttering (Kemerdere et al., 2016). Post-operative damage to FAT can cause a transient problem of initiating spontaneous speech, although this problem often resolves itself rapidly and almost completely (Vassal et al., 2014; Fujii et al., 2015; Kinoshita et al., 2015; Young et al., 2020).

More specifically, a link has been established between fluency in speech production and FAT using different techniques and populations. In a study of 35 individuals with PPA, Catani et al. (2013) did not find a general correlation between FAT measures and overall language impairment, grammatical impairment, repetition, or word comprehension, but they reported that FAT abnormalities are particularly correlated with agrammatic PPA. Moreover, FA of FAT was positively correlated with fluency, measured as words per minute, and mean length of utterance, while radial diffusivity showed an inverse correlation with the same two measures (see also Mandelli et al., 2014). Similarly, in a study of 46 chronic post-stroke individuals, Alyahya et al. (2020) found that the properties of FAT and the anterior parts of AF were predictive of both the quantity and quality of the connected speech (see also Basilakos et al., 2014; Halai et al., 2017; Ille et al., 2018). In yet another population, Chenausky et al. (2017) tested 10 minimally verbal children with Autism Spectrum Disorder (ASD), and found a correlation between FA in AF and FAT in the percentage of correct syllable-initial consonants and percentage of syllable-insertion errors, respectively.

FAT abnormalities have also been linked to stuttering (Kronfeld-Duenias et al., 2016). The nature of the findings has been different though. While Kornfeld-Duenias and colleagues found different diffusivity rates in the left FAT to be predictive of stuttering, Neef et al. (2018) found stronger connectivity of the right FAT to be predictive of more severe stuttering. Consequently, the interpretations were different: the former highlighted the critical importance of left FAT for linguistic sequencing, while the latter took their findings to imply an amplification of the function of the right IFG, namely enforcing global inhibition. While a contribution is clear, more work is needed to understand the role of the left vs. right FAT in stuttering. More recently, FAT has been linked to articulatory-motor planning (Faulkner and Wilshire, 2020; although the use of a probabilistic map in that study makes tract localization less reliable). As noted in the earlier sections, disentangling phonological and post-phonological processes in language production can be tricky, but at least one study that has specifically tested the contribution of FAT to speech apraxia in 52 stroke survivors found apraxia to be associated with lesions to the pre- and post-central gyri and the left dorsal AF but not with FAT (Chenausky et al., 2020).

It is worth mentioning that the FAT is often implicated in verbal fluency tasks along with other tracts, especially IFOF, although the evidence is, as in the other cases, far from convergent. Some studies have reported a correlation with both category and letter fluency scores (Li et al., 2017; Blecher et al., 2019; Costentin et al., 2019; Sanvito et al., 2020), some only with letter/phoneme fluency (Cipolotti et al., 2016; Keser et al., 2020), some with morpheme-based fluency (Yablonski et al., 2021) and some with none of these (Tseng et al., 2019; Vallesi and Babcock, 2020). Costentin et al. (2019) found a correlation between verbal fluency scores and lesions to FAT and a number of other tracts in 48 individuals with Parkinson’s Disease. However, the decline in performance in these tasks after surgery was not correlated with the proportion of the fibers or the number of tracts disconnected. In short, the same heterogeneity observed in the correspondence between verbal fluency scores and some of the other white matter tracts is evident here as well.

Moreover, and similar to the SLF and AF, a syntactic function has also been proposed for FAT, mainly due to its connection to IFG, which has, by some, been proposed as a critical region for syntactic production (e.g., Friederici et al., 2003). The evidence for this link, however, is not watertight. For example, the evidence linking agrammatic aphasia to FAT abnormalities (e.g., Catani et al., 2013) is often confounded with other problems, e.g., phonological planning for longer utterances and higher demands on working memory. Incidentally, some researchers have linked bilateral FAT to working memory in older adults (Rizio and Diaz, 2016). A study that is often taken as a clear evidence in favor of a syntactic role for FAT is the study of Sierpowska et al. (2015). These authors reported a case of intraoperative stimulation of the left FAT, where the patient showed a selective deficit for generating verbs by adding morphemes to nouns (e.g., book → booked) instead of producing the verb usually associated with the noun (book → read). This was taken as a marker of a deficit specific to morphological processing. However, the task has a strong cognitive control demand (rejecting a high-frequency, strongly associated verb in favor of a lower-frequency, weakly associated alternative), typical for left IFG recruitment (e.g., Thompson-Schill et al., 1997). It is thus possible that FAT’s role is related to conflict resolution.

In line with this hypothesis are two other reports of intraoperative stimulations, by Chernoff et al. (2018) and Dragoy et al. (2020). In the former study, the authors reported a patient with surgical damage to the connectivity of the left FAT, who showed a selective post-surgical impairment of fluency in the form of difficulty with voluntary speech and more complex sequences, while remaining unimpaired in production tasks such as picture naming and auditory repetition. In the latter, Dragoy and colleagues reported that cortical stimulation of the termination points of the left FAT (superiorly in the SMA and pre-SMA and inferiorly in the pars triangularis and opercularis of IFG) caused selective impairment in a sentence completion task with low close probability (e.g., sentence prompts such as “A piggy is chewing…” which can be completed with a number of different words). Past research has shown a similar involvement of the IFG (and potentially ACC, SMA, and pre-SMA) in high-conflict tasks and those with under-determined responses, such as spontaneous speech or completing sentences with many possible endings (e.g., Robinson et al., 2010).

These results bring up the question of whether the FAT is truly involved in “syntactic” processing or rather in conflict resolution operations that are often required for the processing of syntactically complex structures. Chernoff et al. (2019) proposed an alternative syntactic function for the tract, namely, sequencing complex actions with a hierarchical structure. In a case study of a patient undergoing awake craniotomy for removing a left frontal tumor, the authors showed that electrical stimulation of the left FAT affected pauses at the beginning of grammatical phrases without influencing either word durations or the durations of noun phrases. This is a neat and informative finding, but it does not necessarily mean involvement in any complex syntactic function. In section 2, under *Articulatory processing*, we reviewed the role of the planning loop, consisting of pre-SMA and left pIFS, which buffers utterances before it is time to send them to the SMA, vPMC, and ultimately vMC for articulation. It is reasonable to assume that buffering happens at the level of short grammatical phrases, and to the extent that FAT is involved in the transmission of these buffered chunks, it is expected that the stimulation effects should manifest at phrasal boundaries. But note that this is a lower-level sequencing operation for motor execution, rather than a higher-level syntactic operation, as in generating a hierarchical syntactic structure *per se*.

A closer look at the right FAT may help adjudicate some of these competing representations. The Right IFG (rIFG) has long been proposed as a critical pathway in “stopping” behavior (Aron et al., 2003, 2004; Aron, 2007). Through the direct pathway, rIFG activates the subthalamic nucleus to enforce stopping (Aron and Poldrack, 2006; Cai and Leung, 2009; Favre et al., 2013; Jahanshahi, 2013; Wiecki and Frank, 2013; Obeso et al., 2014; van Wouwe et al., 2017). There is now evidence that pre-SMA may be a part of this pathway (Nachev et al., 2008; Aron et al., 2016). The right pre-SMA is usually more activated during successful than unsuccessful stops (Aron and Poldrack, 2006; Aron, 2007; Boehler et al., 2010). Its lesions cause a deficit in the execution of complex motor movements, especially in the presence of competing action plans (Nachev et al., 2007), and its direct stimulation stops ongoing movement (Lüders et al., 1988; Mikuni et al., 2006). Whether specialized for “stopping” or rather, context monitoring (Hampshire et al., 2010; Chatham et al., 2012; Erika-Florence et al., 2014; Hampshire, 2015), the evidence strongly points to the involvement of these regions, and their connecting fiber right FAT, in inhibitory control of behavior. Given this evidence, and the symmetry of the cortico-basal ganglia-thalamic-cerebellar circuits in the two hemispheres, Dick et al. (2019) proposed that the FAT is involved in the same function, namely selecting the appropriate plan for motor actions among competing alternatives, in both hemispheres. On the left side, this function is primarily—but not exclusively (Budisavljevic et al., 2017)—applied to language (see Nozari and Hepner, 2019a,b, for a potential application to decisions regarding stopping or proceeding in language production). On the right side, the function applies more broadly to the action domain, especially in visuo-motor tasks. Such a gating function is highly appealing from the perspective of domain-general computations applied to domain-specific representations (e.g., Middleton and Strick, 2000; Hepner and Nozari, 2019), and is a promising framework for future studies of FAT.

### 11.3. Summary

Due to its links to IFG, pre-SMA, and SMA, the FAT is a good candidate for implementing domain-general functions that also apply to language processing. Although both motor and syntactic functions have been proposed, a broader consideration of the role of the regions connected by this tract implicates it in monitoring and control processes that, especially on the left side, regulate the chunking and outputting of articulatory segments. Future work can further test the scope and limits of these functions in FAT.

## 12. Summary and recommendations for future directions

Table 1 provides a summary of the language-related functions attributed to the eight major tracts reviewed above. Generally speaking, the operations linked to the tracts reviewed in the earlier sections of this article are compatible with the architecture of the dual-stream model. The ventral tracts are involved primarily in mapping the auditory input to lexical and ultimately semantic representations in comprehension, as well as mapping semantic concepts onto lexical representations in production. On the other hand, the dorsal tracts are primarily involved in more distal operations in the production pathway, i.e., mapping phonological representations to articulatory plans, buffering of those plans, and mapping them onto their corresponding articulatory motor outputs. These tracts also carry out the mapping between auditory representations and the production chain mentioned above and are thus critical in auditory repetition tasks. Past this rough characterization, however, there are several as-of-yet-unknown details. Below, we discuss some of the outstanding issues (Box 1), and some recommendations for addressing them in future research.

**Table 1:** Summary of the language-related functions attributed to the eight major tracts reviewed in this article.

Function	Tract	References	Method	Population
**Generally implicated in comprehension**	*Left ILF*	Del Tufo et al. (2019)	DTI	TDC (6–10 yrs.)
		Griffis et al. (2017)	VLSM	A
		Ivanova et al. (2016)	DTI	A
		Turken and Dronkers (2011)	DTI, fMRI	NA
		Zhang et al. (2018)	VLSM	A
	*Left IFOF*	Griffis et al. (2017)	VLSM	A
		Turken and Dronkers (2011)	DTI, fMRI	NA
		Zhang et al. (2018)	VLSM	A
	*Left UF*	Catani et al. (2013)	DTI	PPA
		Dodson et al. (2018)	dMRI	TDC (6 yrs.)
		Fridriksson et al. (2013)	DTI	A
	*Right* *UF*	Dodson et al. (2018)	dMRI	TDC (6 yrs.)
	*Left EmC*	Kourtidou et al. (2021)	DTI	A
		Kümmerer et al. (2013)	VLBM	S
		Rolheiser et al. (2011)	DTI	S, NA
		Verly et al. (2019)	DTI	CWDLD, TDC
		Wong et al. (2011)	DTI	NA
	*Right EmC*	Kourtidou et al. (2021)	DTI	A
	*Left MdLF*	Luo et al. (2020)	DTI	PPA, NA
		Saur et al. (2008)	DTI, fMRI	NA
	*Left AF*	Ivanova et al. (2021)	DTI	A
		Turken and Dronkers (2011)	DTI, fMRI	NA
	*Right AF*	Broce et al. (2015)	DWI	TDC (5–8 yrs.)
	*Right FAT*	Broce et al. (2015)	DWI	TDC (5–8 yrs.)
**Generally implicated in production**	*Left ILF*	Ivanova et al. (2016)	DTI	A
		Tuncer et al. (2021)	DTI	BT
	*Left IFOF*	Dávolos et al. (2020)	DTI	NA
		Grossman et al. (2013)	DTI, VBM	A
		Ivanova et al. (2016)	DTI	A
		Tuncer et al. (2021)	DTI	BT
	*Right UF*	Dodson et al. (2018)	dMRI	TDC (6 yrs.)
	*Left SLF*	Asaridou et al. (2017)	MRI, DTI	TDC (8–10 yrs.)
		Kyeong et al. (2019)	DTI	S
		Maldonado et al. (2011)	IS	BT
		Urger et al. (2015)	DTI	TDC (5–17 yrs.)
	*Left AF*	Gajardo-Vidal et al. (2021)	MRI	S
		Liégeois et al. (2013)	DTI	TBI
	*Left FAT*	Faulkner and Wilshire (2020)	DTI	BT, NA
		Fujii et al. (2015)	IS, DTI	BT
		Kinoshita et al. (2015)	IS, DTI	BT
		Vassal et al. (2014)	DTI, fMRI	NA
		Young et al. (2020)	DTI	BT
**Semantic processing**	*Left ILF*	Harvey and Schnur (2015)	DTI, VLSM	A
	*Left IFOF*	de Zubicaray et al. (2011)	DTI, VBM	NA
		Han et al. (2013)	DTI	BD
		Mirman et al. (2015)	VLSM	A
		Moritz-Gasser et al. (2013)	IS	BT
		Sierpowska et al. (2019)	DTI, IS, VLSM	BT
		Surbeck et al. (2020)	DTI	Sch, NA
	*Right IFOF*	Herbet et al. (2017b)	IS	BT
		Surbeck et al. (2020)	DTI	Sch, NA
	*Left UF*	de Zubicaray et al. (2011)	DTI, VBM	NA
		Mirman et al. (2015)	VLSM	A
		Surbeck et al. (2020)	DTI	Sch, NA
**Lexical-semantic retrieval in comprehension**	*Left ILF*	Griffis et al. (2017)	VLSM	A s
		Harvey and Schnur (2015)	DTI, VLSM	A
	*Left IFOF*	Griffis et al. (2017)	VLSM	A
		Han et al. (2013)	DTI	BD
		Mirman et al. (2015)	VLSM	A
		Sierpowska et al. (2019)	DTI, IS, VLSM	BT
	*Left UF*	Han et al. (2013)	DTI	BD
		Mirman et al. (2015)	VLSM	A
		Zhang et al. (2018)	VLSM	A
		Catani et al. (2013)	DTI	PPA
		Fridriksson et al. (2013)	DTI	A
	*Left EmC*	Rolheiser et al. (2011)	DTI	S, NA
	*Left MdLF*	Luo et al. (2020)	DTI	PPA, NA
	*Left AF*	Ivanova et al. (2021)	DTI	A
		Tanabe et al. (1987)	CT	A
**Semantic-lexical retrieval in production**	*Left ILF*	Fridriksson et al. (2013)	DTI	A
		Griffis et al. (2017)	VLSM	A
		Herbet et al. (2019)	IS	BT
		Jarret et al. (2022)	fMRI, dMRI	NA
		McKinnon et al. (2018)	DKI	S
		Moritz-Gasser et al. (2013)	IS	BT
		Powers et al. (2013)	DTI	PPA
		Raffa et al. (2016)	DTI, nTMS	BT
		Sierpowska et al. (2019)	DTI, IS, VLSM	BT
		Stark et al. (2019)	VLSM	S
	*Left IFOF*	Duffau et al. (2008)	IS	BT
		Duffau et al. (2009)	IS	BT
		Faulkner and Wilshire (2020)	DTI	BT, NA
		Fernández et al. (2020)	DTI, dissection, IS	Postmortem brains, BT, NA
		Gil-Robles et al. (2013)	DTI, fMRI	BT
		Griffis et al. (2017)	VLSM	A
		Han et al. (2013)	DTI	BD
		Harvey and Schnur (2015)	DTI, VLSM	A
		Jarret et al. (2022)	fMRI, dMRI	NA
		Leclercq et al. (2010)	IS, DTI	BT
		Mandonnet et al. (2007)	IS, MRI	BT
		Moritz-Gasser et al. (2013)	IS	BT
		Motomura et al. (2018)	IS	BT
		Raffa et al. (2016)	DTI, nTMS	BT
		Sierpowska et al. (2019)	DTI, IS, VLSM	BT
		Stark et al. (2019)	VLSM	S
	*Left UF*	Fridriksson et al. (2013)	DTI	A
		Han et al. (2013)	DTI	BD
		Jarret et al. (2022)	fMRI, dMRI	NA
		Nomura et al. (2013)	MRI, IS	BT
		Powers et al. (2013)	DTI	PPA
		Raffa et al. (2016)	DTI, nTMS	BT
		Zhang et al. (2018)	VLSM	A
	*Left EmC*	Duffau et al. (2005)	IS	BT
		Jarret et al. (2022)	fMRI, dMRI	NA
		Rolheiser et al. (2011)	DTI	S, NA
	*Left MdLF*	Jarret et al. (2022)	fMRI, dMRI	NA
		Luo et al. (2020)	DTI	PPA, NA
	*Left SLF*	Hillis et al. (2018)	PSLM, DWI	S
		Kyeong et al. (2019)	DTI	S
		McKinnon et al. (2018)	DKI	S
		Powers et al. (2013)	DTI	PPA
		Stamatakis et al. (2011)	DTI	NA
	*Right SLF*	Stamatakis et al. (2011)	DTI	NA
	*Left AF*	Duffau et al. (2002)	IS	BT
		Ivanova et al. (2021)	DWI	A
		Marchina et al. (2011)	DTI	S
		Tanabe et al. (1987)	CT	A (conduction)
	*Left FAT*	Vallesi and Babcock (2020)	DTI	NA
**Proper noun naming**	*Left UF*	Lauro et al. (2010)	UF removal	BT
		Papagno et al. (2016)	DTI	BT
		Papagno et al. (2011)	DTI, fMRI	BT
**Lexical-semantic control**	*Left ILF*	Dávolos et al. (2020)	DTI	NA
		Harvey and Schnur (2015)	DTI, VLSM	A
		Nugiel et al. (2016)	DTI	NA
	*Left IFOF*	Harvey and Schnur (2015)	DTI, VLSM	A
		Nugiel et al. (2016)	DTI	NA
	*Right IFOF*	Dávolos et al. (2020)	DTI	NA
	*Left UF*	Di Tella et al. (2020)	DTI	PD, NA
		Harvey et al. (2013)	DTI, fMRI	A, NA
	*Right UF*	Di Tella et al. (2020)	DTI	PD, NA
**Phonological processing**	*Left SLF*	Dodson et al. (2018)	dMRI	TDC (6 yrs.)
		Han et al. (2016)	DTI	BD
		Kyeong et al. (2019)	DTI	S
		McKinnon et al. (2018)	DKI	S
		Travis et al. (2017)	DTI	TDC (5.10–6.10 yrs.)
	*Left AF*	Giampiccolo et al. (2020)	DTI, rTMS,	BT
		Jarret et al. (2022)	fMRI, dMRI	NA
		Mandonnet et al. (2007)	IS, MRI	BT
		Schwartz et al. (2012)	VLSM	A
		Tanabe et al. (1987)	CT	A
		Yamada et al. (2007)	DWI	A (conduction)
**Auditory repetition**	*Left EmC*	Kourtidou et al. (2021)	DTI	A
	*Left MdLF*	Saur et al. (2008)	DTI, fMRI	NA
	*Left SLF*	Breier et al. (2008)	DTI	S
		Kyeong et al. (2019)	DTI	S
	*Left AF*	Breier et al. (2008)	DTI	S
		Forkel et al. (2020)	DTI	PPA, NA
		Kim and Jang (2013)	DTI	A, NA
		Saur et al. (2008)	DTI, fMRI	NA
		Shinoura et al. (2013)	DTI	BT
		Sierpowska et al. (2017)	IS	BT
		Tanabe et al. (1987)	CT	A
		Yamada et al. (2007)	DWI	A (conduction)
**Auditory processing**	*Left MdLF*	Tremblay et al. (2019)	DTI	NA
		Wong et al. (2011)	DTI	NA
	*Right MdLF*	Tremblay et al. (2019)	DTI	NA
	*Left AF*	Li et al. (2021)	DTI	BT
		Oechslin et al. (2010)	DTI	NA
		Tremblay et al. (2019)	DTI	NA
		Vaquero et al. (2021)	DTI, EEG	NA
	*Right AF*	Tremblay et al. (2019)	DTI	NA
	*Left FAT*	Sihvonen et al. (2021)	DTI	S
**Reading**	*Left ILF*	Arrington et al. (2017)	DTI	School-aged TDC with normal and poor phonological abilities
		Broce et al. (2019)	DTI	TDC (5–8 yrs.)
		Carter et al. (2009)	DTI	CWDDL, TDC
		Enatsu et al. (2017)	DTI, IS	Ep
		Epelbaum et al. (2008)	DTI, fMRI, VBM	Ep
		Farah et al. (2020)	DTI	Pre-school TDC
		Gil-Robles et al. (2013)	DTI, fMRI	BT
		Grotheer et al. (2021)	fMRI, dMRI, qMRI	NA
		Horowitz-Kraus et al. (2014)	DTI	TDC (adolescents)
		Motomura et al. (2014)	DTI, IS	BT
		Sarubbo et al. (2015)	DTI, IS	BT
		Steinbrink et al. (2008)	DTI, VBM	Adults with impaired reading/spelling
		Su et al. (2018a)	DWI	CWDLD, TDC
		Vanderauwera et al. (2017)	DTI	Pre-reading children with and without a familial risk for dyslexia (5–6 yrs.)
		Wang et al. (2020)	DWI	BD
	*Right ILF*	Carter et al. (2009)	DTI	CWDLD, TDC (10–14 yrs.)
		Horowitz-Kraus et al. (2014)	DTI	TDC (adolescents)
	*Left IFOF*	Arrington et al. (2017)	DTI	School-aged TDC with normal and poor phonological abilities
		Broce et al. (2019)	DTI	TDC (5–8 yrs.)
		Grotheer et al. (2021)	fMRI, dMRI, qMRI	NA
		Kumar and Padakannaya (2019)	DTI, fMRI	NA
		Steinbrink et al. (2008)	DTI, VBM	Adults with impaired reading/spelling
		Vanderauwera et al. (2018)	DWI	TDC (5–6 years)
		Vanderauwera et al. (2017)	DTI	Pre-reading children with and without a familial risk for dyslexia (5–6 yrs.)
		Vandermosten et al. (2015)	DWI	Pre-reading children with and without a familial risk for dyslexia (5–6 yrs.)
		Zhao et al. (2016)	DTI	CWDLD, TDC
	*Right IFOF*	Broce et al. (2019)	DTI	TDC (5–8 yrs.)
		Farah et al. (2020)	DTI	Pre-school TDC
	*Left VOT*	Broce et al. (2019)	DTI	TDC (5–8 yrs.)
		Grotheer et al. (2021)	fMRI, dMRI, qMRI	NA
	*Right VOT*	Broce et al. (2019)	DTI	TDC (5–8 yrs.)
	*Left UF*	Arrington et al. (2017)	DTI	School-aged TDC with normal and poor phonological abilities
		Bakhtiari et al. (2014)	DTI	NA
		Cummine et al. (2015)	DTI	NA
		Welcome and Joanisse (2014)	DTI	NA
	*Right UF*	Arrington et al. (2017)	DTI	School-aged TDC with normal and poor phonological abilities
		Bakhtiari et al. (2014)	DTI	NA
	*Left SLF*	Bakhtiari et al. (2014)	DTI	NA
		Borchers et al. (2019)	DTI	TDC (5.10–6.10 yrs.)
		Bruckert et al. (2019)	DTI	Children, born full- term and preterm
		Travis et al. (2017)	DTI	TDC (5.10–6.10 yrs.)
	*Right SLF*	Bakhtiari et al. (2014)	DTI	NA
		Borchers et al. (2019)	DTI	TDC (5.10–6.10 yrs.)
		Bruckert et al. (2019)	DTI	Children, born full- term and preterm
	*Left AF*	Dodson et al. (2018)	dMRI	TDC (6 yrs.)
		Gullick and Booth (2014)	DTI	TDC (8–14 yrs.)
		Gullick and Booth (2015)	DTI	TDC (8–14 yrs.)
		Hoeft et al. (2011)	DTI, fMRI	CWDLD, TDC
		Saygin et al. (2013)	DTI	TDC (4–6 yrs.)
		Thiebaut de Schotten et al. (2014)	DTI, fMRI	Illiterate and literate NA
		Vanderauwera et al. (2017)	DTI	Pre-reading children with and without a familial risk for dyslexia (5–6 yrs.)
		Vandermosten et al. (2015)	DWI	Pre-reading children with and without a familial risk for dyslexia (5–6 yrs.)
		Yeatman et al. (2011)	DTI	TDC (7–11 yrs.)
	*Right AF*	Hoeft et al. (2011)	DTI, fMRI	CWDLD, TDC
		Vanderauwera et al. (2017)	DTI	Pre-reading children with and without a familial risk for dyslexia (5–6 yrs.)
**Syntactic processing**	*Left EmC*	Griffiths et al. (2012)	DTI	S, NA e
		Papoutsi et al. (2011)	DTI, fMRI	S, NA
		Rolheiser et al. (2011)	DTI	S, NA
	*Left SLF*	Mills et al. (2013)	DTI	Children with high functioning autism, TDC
		Vidorreta et al. (2011)	IS	BT
		Wilson et al. (2011)	VBM, DTI	PPA
	*Right SLF*	Mills et al. (2013)	DTI	Children with high functioning autism, TDC
	*Left AF*	Mills et al. (2013)	DTI	Children with high functioning autism, TDC
		Papoutsi et al. (2011)	DTI, fMRI	S, NA
	*Left FAT*	Catani et al. (2013)	DTI	PPA
		Chernoff et al. (2019)	IS, DTI	BT, NA
		Chernoff et al. (2018)	DTI, fMRI	BT
		Dragoy et al. (2020)	IS, DTI	BT
		Mandelli et al. (2014)	DTI	PPA, NA
		Yablonski et al. (2021)	DTI, fMRI	NA
**Language-related working memory**	*Left EmC*	Lopez-Barroso et al. (2011)	DTI	NA
	*Left SLF*	Peters et al. (2012)	DTI	TDC, NA (8–21 yrs.)
	*Right SLF*	Peters et al. (2012)	DTI	TDC, NA (8–21 yrs.)
	*Left FAT*	Rizio and Diaz, 2016	DTI	NA
	*Right FAT*	Rizio and Diaz, 2016	DTI	NA
		Varriano et al. (2018)	DTI	NA
		Varriano et al. (2020)	VBM	NA
	*Left AF*	Teubner-Rhodes et al. (2016)	DTI	NA
**Speech fluency**	*Left SLF*	Spinelli et al. (2015)	VBM	PPA, NA
	*Left EmC*	Efthymiopoulou et al. (2017)	CT, MRI	A
		Kourtidou et al. (2021)	DTI	A
	*Left AF*	Basilakos et al. (2014)	MRI	A
		Chenausky et al. (2020)	VLSM	A
		Fridriksson et al. (2013)	DTI	A
		Halai et al. (2017)	DTI	S
		Ivanova et al. (2021)	DTI	A
		López-Barroso et al. (2013)	DTI, fMRI	NA
		Marchina et al. (2011)	DTI	S
	*Left FAT*	Basilakos et al. (2014)	MRI	A
		Catani et al. (2013)	DTI	PPA
		Chenausky et al. (2017)	DTI	TDC (3.5–9.8 yrs.)
		Chenausky et al. (2020)	VLSM	A
		Halai et al. (2017)	DTI	S
		Ille et al. (2018)	DTI, rTMS	BT
		Jarret et al. (2022)	fMRI, dMRI	NA
		Kemerdere et al. (2016)	IS, DTI	BT
		Kronfeld-Duenias et al. (2016)	DTI	St, NA
		Mandelli et al. (2014)	DTI	PPA, NA
		Neef et al. (2018)	DTI, fMRI	St, NA
	*Right FAT*	Chenausky et al. (2017)	DTI	TDC (3.5–9.8 yrs.)
		Chenausky et al. (2020)	VLSM	A
		Neef et al. (2018)	DTI, fMRI	St, NA
		Spinelli et al. (2015)	VLSM	PPA, NA
**Semantic fluency**	*Left ILF*	Griffis et al. (2017)	VLSM	A
	*Left IFOF*	Almairac et al. (2015)	VLSM	A
		Gonzalez et al. (2021)	DTI	TDC (7–13 yrs.)
		Griffis et al. (2017)	VLSM	A
	*Left UF*	Di Tella et al. (2020)	DTI	PD, NA
		Lauro et al. (2010)	UF removal	BT
		Li et al. (2017)	DTI	S, NA
		Papagno et al. (2016)	DTI	BT
		Papagno et al. (2011)	DTI, fMRI	BT
		Powers et al. (2013)	DTI	PPA
		Rodríguez-Aranda et al. (2016)	DTI, VBM	AD, NA
	*Right UF*	Rodríguez-Aranda et al. (2016)	DTI, VBM	AD, NA
	*Left SLF*	Gonzalez et al. (2021)	DTI	TDC (7–13 yrs.)
		Powers et al. (2013)	DTI	PPA
		Pustina et al. (2014)	DTI, VBM	E, NA
	*Right SLF*	Gonzalez et al. (2021)	DTI	TDC (7–13 yrs.)
		Pustina et al. (2014)	DTI, VBM	Ep, NA
	*Left AF*	Blecher et al. (2019)	DTI	MS
		Gonzalez et al. (2021)	DTI	TDC (7–13 yrs.)
		Sanvito et al. (2020)	DTI, fMRI	NA
	*Right AF*	Gonzalez et al. (2021)	DTI	TDC (7–13 yrs.)
	*Left FAT*	Blecher et al. (2019)	DTI	MS
		Costentin et al. (2019)	MRI	PD
		Li et al. (2017)	DTI	S, NA
		Sanvito et al. (2020)	DTI, fMRI	NA
	*Right FAT*	Blecher et al. (2019)	DTI	MS
**Phoneme/letter fluency**	*Left UF*	Kljajevic et al. (2016)	DTI	NA
		Li et al. (2017)	DTI	S, NA
		Papagno et al. (2011)	DTI, fMRI	BT
		Serra et al. (2012)	DTI	Various forms of dementia, NA
	*Left SLF*	Gonzalez et al. (2021)	DTI	TDC (7–13 yrs.)
		Madhavan et al. (2014)	DTI	NA
		Pustina et al. (2014)	DTI, VBM	Ep, NA
	*Right SLF*	Pustina et al. (2014)	DTI, VBM	Ep, NA
	*Left AF*	Blecher et al. (2019)	DTI	MS
		Sanvito et al. (2020)	DTI, fMRI	NA
	*Right AF*	Gonzalez et al. (2021)	DTI	TDC (7–13 yrs.)
	*Left FAT*	Blecher et al. (2019)	DTI	MS
		Cipolotti et al. (2016)	PLSM	BD
		Costentin et al. (2019)	MRI	Par
		Keser et al. (2020)	DTI	MS
		Li et al. (2017)	DTI	S, NA
		Sanvito et al. (2020)	DTI, fMRI	NA
	*Right FAT*	Keser et al. (2020)	DTI	MS

*A, aphasia; AD, Alzheimer’s disease; BD, brain damage (broadly defined, encompassing multiple categories); BT, brain tumor; CT, computerized tomography; CWDLD, children with developmental language disorders; DKI, diffusional kurtosis imaging; dMRI, diffusion magnetic resonance imaging; DTI, diffusion tensor imaging; DWI, diffusion-weighted imaging; Ep, epilepsy; fMRI, functional magnetic resonance imaging; IS, intraoperative stimulation; MS, multiple sclerosis; NA, neurotypical adults; nTMS, navigated transcranial magnetic stimulation; PD, Parkinson disease; PLSM, parcel based lesion symptom mapping; PPA, primary progressive aphasia; qMRI, quantitative magnetic resonance imaging; S, stroke; Sch, schizophrenia; St, stuttering; TBI, traumatic brain injury; TDC, typically developing children; VBM, voxel-based morphometry; VLSM, voxel-based lesion symptom mapping; yrs., years*.

Box 1 **Outstanding questions for future research.**

*Ventral tracts*

(1)  Are some tracts specialized for comprehension vs. production?

(2)  Are some tracts specialized for semantic control?

*Dorsal tracts*

(1)  Which tracts are specialized for phonological buffering in working memory?

(2)  What is the role of FAT?

*Ventral and dorsal tracts*

(1)  Which tracts are involved in which aspects of reading?

(2)  Which tracts are involved in which aspects of syntactic processing?

### 12.1. Ventral stream

Two outstanding questions are (1) Are some tracts specialized for comprehension vs. production?; and (2) Are some tracts specialized for semantic control as opposed to semantic activation? Currently, the empirical data are not conclusive on these two points. Although there is some evidence that IFOF and ILF may be more involved in production and perception, respectively, this distinction is not uncontested. The evidence linking individual tracts to semantic-lexical control is even less consistent, with ILF, IFOF, and UF each implicated in some, but not in other, studies. Thus, future research on ventral tracts can benefit from cognitive tasks that: (a) compare production vs. comprehension using a similar set of materials, e.g., picture naming vs. picture-word matching in individuals with brain damage; and (b) compare production/perception in conditions with low control demands to those with high control demands. An example in production is picture naming in the context of other semantically related vs. unrelated items (Costa et al., 2006; Schnur et al., 2009; Nozari et al., 2016b; Hepner and Nozari, 2020; McDonagh et al., 2020). The comprehension equivalent is picture-word matching with semantically related vs. unrelated distractors (e.g., Nozari, 2019). Although some of these tasks have been used in some studies, there is a clear need for more studies with larger sample sizes, larger sets, and better-controlled materials to reconcile some of the discrepancies in the existing findings.

### 12.2. Dorsal stream

Two outstanding questions here concern the differentiation in the role of the tracts involved in phonological processing, and the role of FAT. We unpack each question below: (1) Which tracts are involved in pure mapping of phonological representations to more peripheral representations, and which tracts are critical in the working memory (i.e., buffering) operations involving phonological representations. This distinction can be behaviorally tested by comparing the production of shorter vs. longer words (which require greater buffering; e.g., Goldrick and Rapp, 2007), or other tests of phonological working memory, although preferably those that do not pose additional demands on conflict resolution, such as discriminating between close phonological alternatives in working memory. Some researchers have proposed that the same regions storing phonological representations are also involved in buffering them (Acheson et al., 2010), while others have suggested separate neural regions specialized for phonological buffering (Yue et al., 2018). The identification of the white matter tracts selectively involved in phonological working memory may shed some light on this debate. (2) The role of FAT is also still debated, with the proposal of a domain-general conflict-resolution and selection function, with some degree of domain-specificity for language in the left hemisphere, as the most promising theoretical framework for designing empirical studies.

### 12.3. Ventral and dorsal streams

Two sets of operations have been frequently suggested for tracts in both streams, reading and syntactic production. It is not surprising that reading has its signature over both tracts, as it can encompass a wide range of operations including the retrieval of phonological, lexical, and semantic representations, conflict resolution for visually, phonologically, or semantically similar items, and in some cases even activating motor commands. Most studies that link reading to different tracts have not disentangled different aspects of reading. Similarly, syntax encompasses a wide range of operations, some but not all of which require maintaining long-distance dependencies and reconciling competition between alternative representations, which may reflect more domain-general abilities rather than syntactic processes *per se* (e.g., Nozari and Omaki, 2022). Most studies of syntactic processing have not attempted to carefully disentangle these facets of processing.

In short, two questions are outstanding here: (1) Which tracts are involved in which aspect(s) of reading? More data from studies that differentiate the contribution of lexical and sublexical reading inspired by cognitive theories (e.g., Coltheart et al., 2001) would shed light on this question. (2) Which tracts are involved in which aspect(s) of syntactic processing? A more systematic study of syntactic processing by carving it at its joints is likely to yield more informative results. One proposed dimension is Matchin et al.’s (2017) separation of syntactic operations in comprehension vs. production. But there are many other possible divisions (or subdivisions within the comprehension/production framework) that can further shed light on the role of different neural regions in various aspects of syntactic processing. For example, one could test whether morphological processing can be disentangled from working memory processes which mediate the relationship between two parts of a sentence with dependencies.

## 13. Conclusion

Much has already been learned about the network involved in processing language production and comprehension, including the white matter pathways that connect various cortical regions. The field has moved beyond the general question of whether a tract is or is not involved in language processing and has reached a state of probing the nuanced nature of such involvement. This is an excellent time for combining theoretically inspired approaches with neural investigations. Specifically, the review above shows the need for moving away from paradigms that confound multiple operations, e.g., verbal fluency tasks, and towards those that can better tease apart cognitive components such as semantic activation vs. semantic control, phonological activation vs. phonological buffering, and pure syntactic operations vs. domain-general processes that support such operations.

## Author contributions

ES contributed to conceptualizing the work, reviewing the literature, generating the table and figures, and writing the masnucript. NN contributed to conceptuliaizing and writing the manuscript. All authors contributed to the article and approved the submitted version.
